# Decitabine increases neoantigen and cancer testis antigen expression to enhance T-cell–mediated toxicity against glioblastoma

**DOI:** 10.1093/neuonc/noac107

**Published:** 2022-04-25

**Authors:** Ruichong Ma, Margarida Rei, Isaac Woodhouse, Katherine Ferris, Sophie Kirschner, Anandhakumar Chandran, Uzi Gileadi, Ji-Li Chen, Mariana Pereira Pinho, Yoanna Ariosa-Morejon, Skirmantas Kriaucionis, Nicola Ternette, Hashem Koohy, Olaf Ansorge, Graham Ogg, Puneet Plaha, Vincenzo Cerundolo

**Affiliations:** MRC Human Immunology Unit, Radcliffe Department of Medicine, University of Oxford, Oxford, UK; Department of Neurosurgery, Oxford University Hospitals NHS Foundation Trust, Oxford, UK; MRC Human Immunology Unit, Radcliffe Department of Medicine, University of Oxford, Oxford, UK; Ludwig Institute for Cancer Research, Nuffield Department of Medicine, University of Oxford, Oxford, UK; MRC Human Immunology Unit, Radcliffe Department of Medicine, University of Oxford, Oxford, UK; Centre for Cellular and Medical Physiology, Nuffield Department of Medicine, University of Oxford, Oxford, UK; MRC Human Immunology Unit, Radcliffe Department of Medicine, University of Oxford, Oxford, UK; Ludwig Institute for Cancer Research, Nuffield Department of Medicine, University of Oxford, Oxford, UK; Ludwig Institute for Cancer Research, Nuffield Department of Medicine, University of Oxford, Oxford, UK; MRC Human Immunology Unit, Radcliffe Department of Medicine, University of Oxford, Oxford, UK; MRC Human Immunology Unit, Radcliffe Department of Medicine, University of Oxford, Oxford, UK; MRC Human Immunology Unit, Radcliffe Department of Medicine, University of Oxford, Oxford, UK; Centre for Cellular and Medical Physiology, Nuffield Department of Medicine, University of Oxford, Oxford, UK; The Jenner Institute, University of Oxford, Oxford, UK; Ludwig Institute for Cancer Research, Nuffield Department of Medicine, University of Oxford, Oxford, UK; Centre for Cellular and Medical Physiology, Nuffield Department of Medicine, University of Oxford, Oxford, UK; The Jenner Institute, University of Oxford, Oxford, UK (Y.A-M., N.T.); MRC Human Immunology Unit, Radcliffe Department of Medicine, University of Oxford, Oxford, UK; Nuffield Department of Clinical Neurosciences, University of Oxford, UK; MRC Human Immunology Unit, Radcliffe Department of Medicine, University of Oxford, Oxford, UK; Department of Neurosurgery, Oxford University Hospitals NHS Foundation Trust, Oxford, UK; Nuffield Department of Clinical Neurosciences, University of Oxford, UK; MRC Human Immunology Unit, Radcliffe Department of Medicine, University of Oxford, Oxford, UK

**Keywords:** decitabine, glioblastoma, immunotherapy, neoantigen, tumor immunology

## Abstract

**Background:**

Glioblastoma (GBM) is the most common and malignant primary brain tumor in adults. Despite maximal treatment, median survival remains dismal at 14–24 months. Immunotherapies, such as checkpoint inhibition, have revolutionized management of some cancers but have little benefit for GBM patients. This is, in part, due to the low mutational and neoantigen burden in this immunogenically “cold” tumor.

**Methods:**

U87MG and patient-derived cell lines were treated with 5-aza-2′-deoxycytidine (DAC) and underwent whole-exome and transcriptome sequencing. Cell lines were then subjected to cellular assays with neoantigen and cancer testis antigen (CTA) specific T cells.

**Results:**

We demonstrate that DAC increases neoantigen and CTA mRNA expression through DNA hypomethylation. This results in increased neoantigen presentation by MHC class I in tumor cells, leading to increased neoantigen- and CTA-specific T-cell activation and killing of DAC-treated cancer cells. In addition, we show that patients have endogenous cancer-specific T cells in both tumor and blood, which show increased tumor-specific activation in the presence of DAC-treated cells.

**Conclusions:**

Our work shows that DAC increases GBM immunogenicity and consequent susceptibility to T-cell responses *in vitro*. Our results support a potential use of DAC as a sensitizing agent for immunotherapy.

Key Points

- DAC increases neoantigen and CTA expression in GBM through hypomethylation
- Increased antigen expression leads to enhanced antigen-specific T-cell–mediated toxicity
- DAC also increases pre-existing autologous tumor-specific T-cell–mediated toxicity.

**Importance of the Study**

Current immunotherapeutic strategies, including checkpoint inhibitor therapy, against GBM have poor efficacy. This is partly due to the low neoantigen burden in GBM. Here, we show for the first time that DAC treatment of cancer cells *in-vitro* increases the expression of neoantigens in addition to a plethora of CTA leading to increased immunogenicity and T-cell–mediated toxicity of this immunologically cold tumor. In addition, we show that treatment of tumor cell lines with DAC *in-vitro* increases activation of pre-existing autologous tumor-specific T cells isolated from GBM patients. These results demonstrate novel immunogenic mechanisms of DAC which could sensitize GBM to immunotherapy. Our study provides a strong rationale to investigate DAC’s synergistic effects with immunotherapeutic drugs in a clinical setting.

Glioblastoma (GBM) is the most common and the most malignant brain tumor in adults. Each year in the UK more than 4000 new cases of central nervous system (CNS) cancers are diagnosed, which equates to around 7 per 100 000 population. Although brain tumors account for less than 2% of all primary tumors they are responsible for 7% of the years of life lost from cancer before age 70 (Office of National Statistics 2006 Series MB1 No. 34). Unfortunately, despite significant research into these tumors, the latest survival trends for patients with CNS malignancies have remained largely static^1^ reflecting the lack of therapeutic options for patients. The current gold standard of treatment involves gross total resection (GTR, as defined by complete resection of contrast-enhancing tumor on a postoperative scan performed within 72 hours of surgery) of the tumor followed by adjuvant radio-/chemotherapy with temozolomide.^2^ Despite optimal treatment the median survival for such patients is still only 14–24 months and a five-year survival of approximately 10%.^2,3^

Immunotherapies, especially checkpoint inhibition, have revolutionized the management of some previously untreatable cancers such as metastatic melanoma,^4–6^ nonsmall cell lung cancer^7–10^ and renal carcinoma,^11,12^ but the response seen in GBM is much more modest.^13–16^ As their use has become more widespread, it is clear that checkpoint inhibitor efficacy is linked to the tumor mutational burden (TMB)^14^ and consequent neoantigen burden.^17,18^ The low TMB seen in GBM,^19,20^ as well as its’ immunosuppressive microenvironment with infiltration of microglia, myeloid-derived suppressor cells, high levels of CCL2, CXCL2, and TGF-ß ^21^ help to explain the poor response rate to such immunotherapies.

Here, we explore the expression of neoantigens and cancer testis antigens (CTA) in GBM. We show that there are potential neoantigen encoding mutations that are not/lowly expressed. Treatment with the DNA methyl transferase inhibitor (DNMTi), 5-aza-2’-deoxycytidine (DAC), can increase the expression of both neoantigen encoding mutations and CTA. Furthermore, we show that increased expression of immunogenic antigens leads to an increased ability for neoantigen- and CTA-specific CD8 cytotoxic T lymphocytes (CTL) to recognize and kill tumor cells *in-vitro* following treatment with DAC.

## Materials and Methods

Detailed experimental methods can be found in the Supplementary Material.

### Ethics Statement

All human samples were collected through the Oxford Brain Bank, approved by the local research ethics committee (15/SC/0639). Specific informed consent was mandatory for each patient and was obtained before each surgery for patients undergoing multiple operations.

### Patients and Human Samples

For each patient, tumor samples and blood were obtained at the time of surgery. Tumor samples were first washed in PBS and mechanically dissociated with removal of blood vessels and necrotic tissue. This material was then digested enzymatically using the human tumor dissociation kit (Miltenyi) as per manufacturer’s instructions. The tumor digests underwent myelin removal and CD45 separation using MACS beads and columns (Miltenyi) as per manufacturer’s instructions. The CD45- fraction was used for establishment of primary tumor cell lines. Peripheral blood mononuclear cells (PBMC) were isolated from the fresh blood by centrifugation on Lymphoprep (Stem Cell Technologies) according to manufacturer’s instructions. HLA haplotyping was performed by the WIMM Sequencing Facility. Patient details and haplotyping information is described in Supplementary Table 5.

### Cell Culture

Primary tumor cell lines were cultured in serum-free media (RHB-A, Takara) supplemented with 20 ng/mL epidermal growth factor, 20 ng/mL fibroblast growth factor (Peprotech), and 100 U/mL penicillin-0.1mg/mL streptomycin (Pen-Strep, Sigma) on laminin-coated flasks. U87MG, HEK293T, and normal human astrocytes (NHA) were cultured in DMEM (Sigma) supplemented with 10% fetal calf serum (FCS, Gibco) and Pen-Strep (D10). Primary immune cells were cultured in RPMI supplemented with 5% human serum (pooled serum from 10 different donors obtained from NHS blood and transplant services), 2mM L-Glutamine, 1x nonessential amino acids, 10mM HEPES, 1mM sodium pyruvate, Pen-Strep and 50 µM 2-mecaptoethanol (RHS5).

### In-Vitro Decitabine Treatment

Cells plated on the previous day were treated with fresh media containing the required concentration of DAC every 24 h, for a total of 48 h. Cells were then cultured with fresh media without decitabine for a further 3 days for transcriptomic analysis or 5 days for proteomic and functional assays.

### Isolation of Neoantigen-Specific T Cells From Patients

Peptide stimulation was performed as previously described.^22^ Neoantigen-specific T cells were isolated using MHC-peptide tetramers as previously described.^23^ CD3/CD8/tetramer+ cells were sorted and further expanded.

### Generation of Neoantigen-Specific T Cells From Healthy Donors

Healthy donor T cells were primed *in-vitro* using a modified version of a previously described technique.^24^ CD3/CD8/tetramer+ cells were sorted and further expanded.

### Mixed Tumor Lymphocyte Culture (MLTC)

Patient PBMC were cocultured with irradiated autologous primary tumor cell line (30Gy) for 10–14 days, in RHS5 supplemented with 10 µg/mL IL-7 and 50 pg/mL IL-12 (Peprotech). The cells were then re-stimulated with additional irradiated tumor cells. Tumor reactivity was measured by intracellular cytokine staining (ICS). Single cells were sorted on either CD3/IFN-γ+, CD3/TNF-α+ or CD3/IFN-γ/TNF-α+ gates for both TCR sequencing and clonal expansion.

### Single Cell Ex-Vivo Expansion of T Cell Clones

Single T-cell clonal expansion was performed by sorting single T cells into 96-well tissue culture plates as described previously.^25^ Clones that had grown sufficiently to be passaged and used for initial screening were then re-stimulated and expanded in RHS5+IL2 and PHA with irradiated feeder cells for further expansion.

### TCR Sequencing and Transduction

Individual or 50 (mini-bulk) T cells were sorted into lysis buffer containing Triton X-100 (Sigma), RNAse inhibitor (Takara), dNTP (NEB), oligo(dT) primer, and TCRα/β-specific primers (IDT). Reverse transcription was performed with SMART Scribe reverse transcriptase (Takara) and a template switch oligo. Total cDNA was amplified by PCR using SeqAmp DNA polymerase (Takara). The PCR product was then used for targeted amplification of the TCRα/β chains in two subsequent rounds of nested PCR, using Phusion High Fidelity DNA polymerase (NEB). One last PCR was performed to add Illumina adaptors. The final library was sequenced using the Miseq Reagent Kit V2 300 cycle in the MiSeq platform (Illumina).

TCRs of interest were cloned into a pHR-SIN plasmid with the mouse TCRα/β constant regions. Amplified TCR chains were purified using magnetic beads (AMPure XP, Beckman Coulter) and cloned in the plasmid using HiFi DNA assembly cloning kit (NEB). Successfully cloned plasmids were confirmed by Sanger sequencing (Source Bioscience).

Lentivirus production was achieved by transfecting HEK 293T cells in 6-well plates with 4.5 µL TurboFectin transfection reagent (Origene), 0.5 µg pCMV-dR8.91, 0.25 µg pMDG-VSVG and 1 µg of pHR-SIN-TCR plasmid. Primary CD8 T cells previously activated during 2 days with CD3/CD28 Dynabeads (Thermo Fisher) in RHS5/2 supplemented with 10ng/mL IL-5 (Peprotech) were incubated with lentivirus and Retronectin (Takara) coated plates.

### T Cell Functional Assays

Cancer cells were plated in RHS5 and, when applicable, pulsed with varying amounts of peptides. Cancer cells were then incubated with MHC-class I blocking antibody (W6/32 at 20 µg/mL), if required. T cells were cocultured with cancer cells at varying effector: target ratios. Cell Stimulation Cocktail (eBioscience) was used to stimulate the positive control cells. Cells were then stained with Zombie Aqua (eBioscience) and antibodies against CD3, CD8, IFN-g, TNFα (Biolegend) using the Intracellular Fixation and Permeabilization Buffer Set (eBioscience) following manufacturer’s instructions and analyzed by flow cytometry.

To evaluate T-cell killing capacity, cells were cocultured for 16h. T-cell killing was assessed by lactate dehydrogenase (LDH) release using the Cyto-tox nonradioactive cytotoxicity assay (Promega) as per manufacturer’s instructions, using an iMark microplate reader (Bio-Rad).

### Flow Cytometry

Data was acquired using Fortessa X-20, X-50 (BD), or Attune NxT (Life technologies) flow cytometers. Cell sorting was performed on either Fusion 2, Aria III (BD) or SH800 (Sony) cell sorters. Data were analyzed using FlowJo (BD). Gating strategy is shown in Supplementary Figure 1.

### Whole Exome Sequencing (WES)

Genomic DNA was extracted from primary tumor samples, cell lines, and PBMC (germ line) using DNEasy blood and tissue kit (Qiagen) as per manufacturer’s instructions. WES was performed by Novogene Co. Ltd using SureSelect Human All Exon V6 (Agilent) and Next Ultra DNA Library Prep Kit (NEB). Data is available on EGA.

### RNA Sequencing

RNA was extracted using RNEasy kit (Qiagen) as per manufacturer’s instructions. 1µg RNA was sent for library preparation and sequencing by Novogene Co. Ltd. Library preparation was performed using Next® Ultra RNA Library Prep Kit for Illumina® (NEB) and sequenced using the NovaSeq platform (Illumina) with a paired-end 150-base-pair sequencing strategy. Data available on EGA.

### Methylation Sequencing

Global methylation analysis was performed by Diagenode using Infinium methylation EPIC BeadChip Kit (Illumina). Analysis was performed using the ChAMP Bioconductor package.^26,27^

For targeted bisulfite sequencing, 2 µg of genomic DNA was bisulfite converted using the Zymo EZ DNA Methylation-Lightning Kit (Cambridge Bioscience, D5030) and loci in the promoter of several genes were specifically amplified using the PyroMark PCR kit (Qiagen, 978703) according to the manufacturer’s recommendations.

### Neoantigen Prediction and Peptides

Neoantigen prediction was performed by an in-house version of MuPeXI^28^ codenamed TUNAPASTA v0.5 which was redeveloped to accept the data format as required, but without any changes to the ranking approach. Predicted neoantigen peptides were bought from Pepscan (Netherlands) and Genecust (France) at crude purity, diluted in DMSO to 10 mg/mL stock, and stored at –80°C.

### Statistical Analysis

Statistical analysis for functional assays and RT-qPCR was performed using Prism 8 (GraphPad) using two-tailed paired student t Test or 2-way analysis of variance (ANOVA) with Tukey’s multiple comparisons test as stated in the results. Results are presented as mean with standard errors. DEseq2,^29^ clusterProfiler^30^ and ChAMP^31^ were utilized mostly with their default parameters, which includes *P* values adjusted using Benjamini Hochberg multiple tests corrections. *P* values: * *P* ≤ .05, ** *P* ≤ .01, ***P ≤ .001, **** *P <* .0001.

## Results

### The Number of Expressed, Nonsilent Mutations is Associated With Survival of GBM Patients

**Fig. 1:**
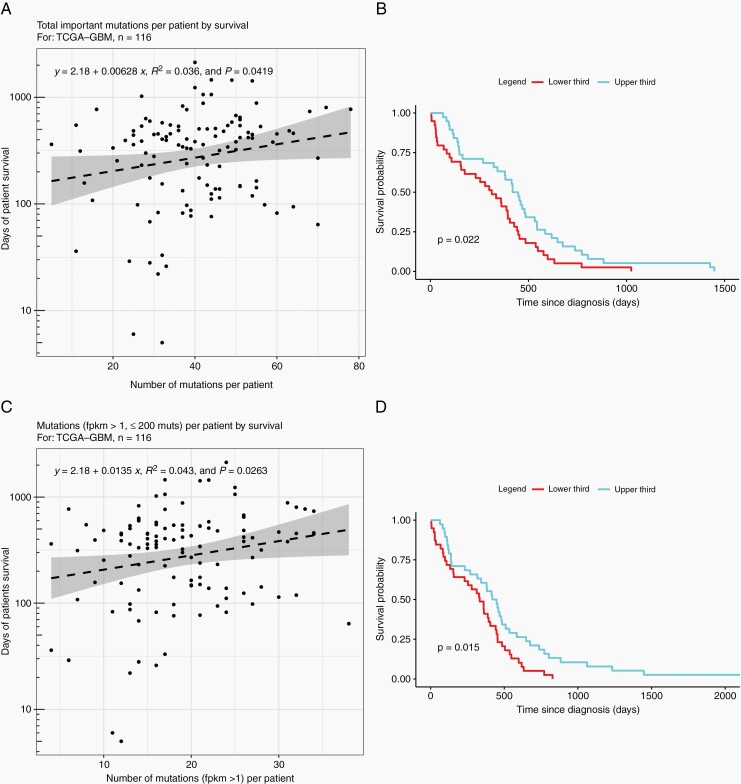
Number of expressed mutations is positively correlated with survival for patients included in TCGA. Scatter plots comparing (A) total important mutations (nonsilent mutations,) and (C) expressed nonsilent mutations against overall survival from diagnosis. Kaplan-Meier plots comparing survival of patients in the upper third and lower third as separated by (B) total number of nonsilent mutations and (D) number of expressed nonsilent mutations.

To explore the effect of number of mutations and their expression, we interrogated the TCGA database for GBM patients with nonhypermutated tumors (>10 and *<*200 mutations), retrieving mutation, transcriptomic, methylation, and survival data for 116 individuals. Analysis of this cohort suggests a positive correlation between survival and number of nonsilent mutations (Figure 1A), and particularly between survival and the number of expressed mutations (FKPM≥1, Figure 1C). In addition, there is a significant improvement in median survival in the top third of patients by both total (Figure 1B) and expressed mutation (Figure 1D) number, compared to those in the bottom third for each group. Looking at methylation of genes harboring nonsilent mutations, genes that are expressed (FKPM*>*1) have a median methylation value of 0.05 compared to 0.32 in genes that are lowly expressed (0<FKPM<1) and 0.56 in genes that are not expressed (FKPM = 0), supporting the rationale to use hypomethylating agents to increase expression of mutated genes (Supplementary Figure 2).

### Decitabine Increases Expression of Potential Neoantigens and CTA

To explore the effect of immunogenic antigen expression in greater detail, we started by analyzing the expression of potential neoantigens on a commercial cell line—U87MG—and primary samples obtained from 4 GBM patients. We utilized mutation data for U87MG, obtained from the Cancer Cell Line Encyclopaedia, and chose the top 9 predicted HLA-A2-restricted neoantigen-encoding mutations (Supplementary Table 4). Analysis of gene expression by RT-qPCR shows that 6/9 genes have little or no expression at baseline (Supplementary Figure 3A). We selected 4 HLA-A2+ patients (Supplementary Table 5) to undergo sequencing of their tumors and their derived cell lines to predict neoantigens. Expression analysis of potential neoantigen-encoding mutations in the cell lines shows that the majority of mutations are either not expressed or lowly expressed (Supplementary Figure 3B). From these, we selected 153 HLA-A2-restricted neoantigens across the 4 patients, for further functional analysis (Supplementary Table 6). Altogether, these data indicate that there are potential neoantigens whose expression could be enhanced, which lead us to investigate the effects of a DNA hypomethylating agent, decitabine.

**Fig. 2:**
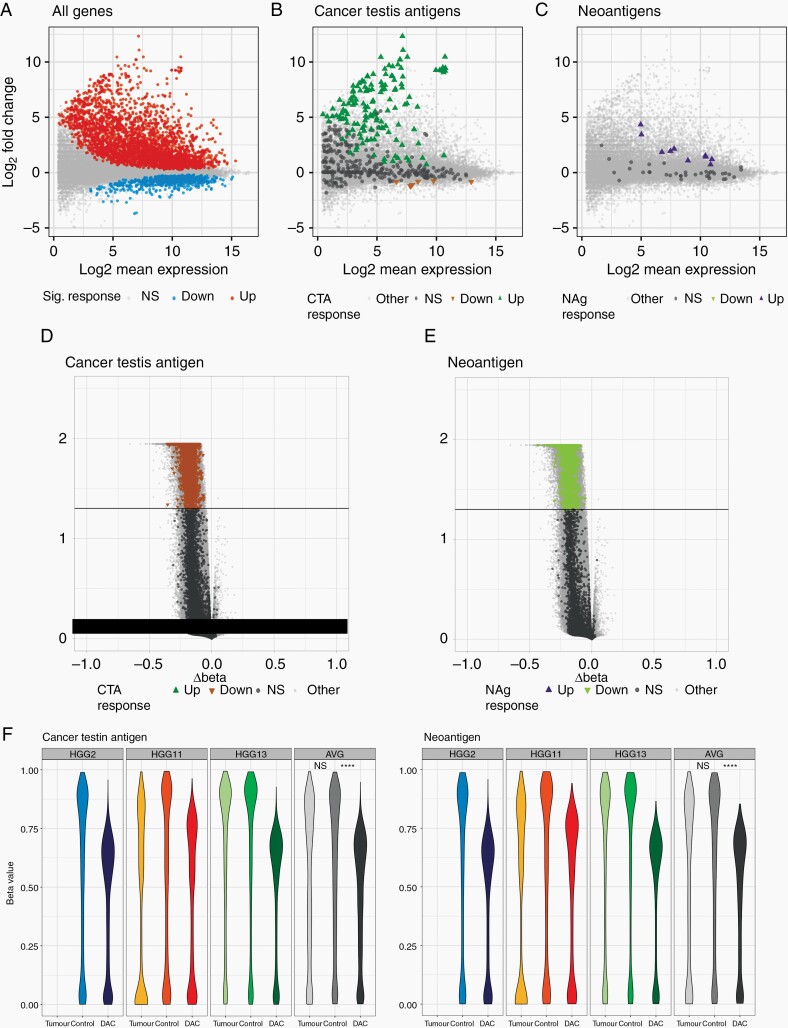
Decitabine decreases methylation and increases expression of both cancer testis antigens and neoantigens in primary GBM cell lines. (A-C) MA plots comparing expression of (A) all genes, (B) cancer testis antigens and (C) neoantigens in primary GBM cell lines with and without DAC treatment. (D,E) Volcano plots showing differential methylation of (D) cancer testis antigens and (E) neoantigens following DAC treatment. Colored dots represent individual genes with significantly (*P <* .05) altered expression following DAC treatment, direction of arrow signifies direction of change. (F,G) violin plots showing methylation levels of (F) cancer testis antigens and (G) neoantigens of primary cell lines with and without DAC treatment as well as primary tumor samples. Data shown are from 3 biological replicates. Tumor—primary tumor sample; control—primary tumor cell lines not treated with DAC; DAC—primary tumor cell lines treated with DAC. * *P <* .05, ** *P <* .01, *** *P <* .001, **** *P <* .0001.

Treatment of cell lines with DAC revealed both a dose-dependent, and treatment duration effect on gene expression with increasing expression following treatment with higher concentrations of DAC and for 48hrs compared to 24hrs (Supplementary Figure 4). RT-qPCR revealed that the expression of 6/9 genes encoding for potential neoantigens in U87MG was significantly upregulated following treatment with 1µM DAC (Supplementary Figure 3A. In addition, targeted next-generation sequencing for selected mutations showed that the mutant: wild-type ratios remained similar or increased (Supplementary Figure 5), addressing any concerns of selective upregulation of the wild-type alleles. We found that changes at the transcriptomic level, at least for the 2 genes tested (NY-ESO and SLC6A12), are reflected at the proteomic level (Supplementary Figure 6). For SLC6A12, this increase in protein is only found in the presence of a proteosome inhibitor. This may reflect misfolding and increased proteasomal turnover of the aberrant protein produced in the presence of the mutation, which is only apparent when proteasomal degradation is inhibited. Transcriptomic analysis of primary patient cell lines showed that there was a generalized increase in transcription following treatment with DAC (Figure 2A, Supplementary Figure 7). We also found that there are potential neoantigens upregulated across all 4 patients (Figure 2C, Supplementary Figure 7). This effect is variable between patients and likely represents differing number of mutations, variable proliferation rates, and differing methylation of genes harboring mutations. The effect on CTA (as determined by a composite of CT database^32^ and da Silva et al.^33^) was even more marked, with this group of genes comprising the majority of the most significantly upregulated genes (Figure 2B, Supplementary Figure 7). We also performed immunopeptidomics to interrogate the change in MHC class I ligandome of HGG13 cell line following treatment with DAC. Unfortunately, we were unable to identify the presentation of any neoantigens in both control and DAC treated cells. This limitation of detection, which was also noted to be the case in a recent clinical trial applying personalized vaccines to patients with GBM,^15^ is not due to the absence of such antigenic peptide sequences in the HLA-associated peptidome but can rather be explained by the limitations in biochemical HLA enrichment efficiency, and the nature of the LC-MS experiment. Interestingly, the cell lines express several immune checkpoint inhibitor ligands including PD-L1 before and after DAC treatment, except 4-1BBL whose expression is upregulated after DAC treatment in 3/4 cell lines (Supplementary Figure 8). In addition, stem/precursor and differentiation markers are generally similar in DAC treated cells compared to control (Supplementary Figure 9) suggesting that the cell lines maintain their phenotypes after DAC treatment. Importantly, although DAC decreased the growth of tumor cells *in -vitro* by day 9, it did not significantly affect the viability of U87MG or primary cell lines HGG2 and HGG13 (Supplementary Figure 10).

We performed global methylation array analysis for three patient samples and showed that, in line with the mechanism of action of DAC, there were 271,567 significantly differentially methylated probes (DMP) from 19,230 genes, all but 1 of which were hypomethylated upon DAC treatment. Of these significantly hypomethylated genes, there were 534 CTA and 110 neoantigens across the 3 patients (Figure 2D,E). Analysis of individual patient samples shows that CTA and neoantigen methylation was significantly lower in the DAC-treated cell lines, confirming that this increase in expression is driven by DAC-induced DNA hypomethylation (Figure 2F,G). Importantly, we observed no DMP between the tumor cell line and tumor sample (Supplementary Figure 11) which indicates that the cell lines established in the lab are a good surrogate of the original patient’s tumor in terms of methylation.

### Isolation of Neoantigen Specific CD8 Cytotoxic T Lymphocytes (CTL)

**Fig. 3:**
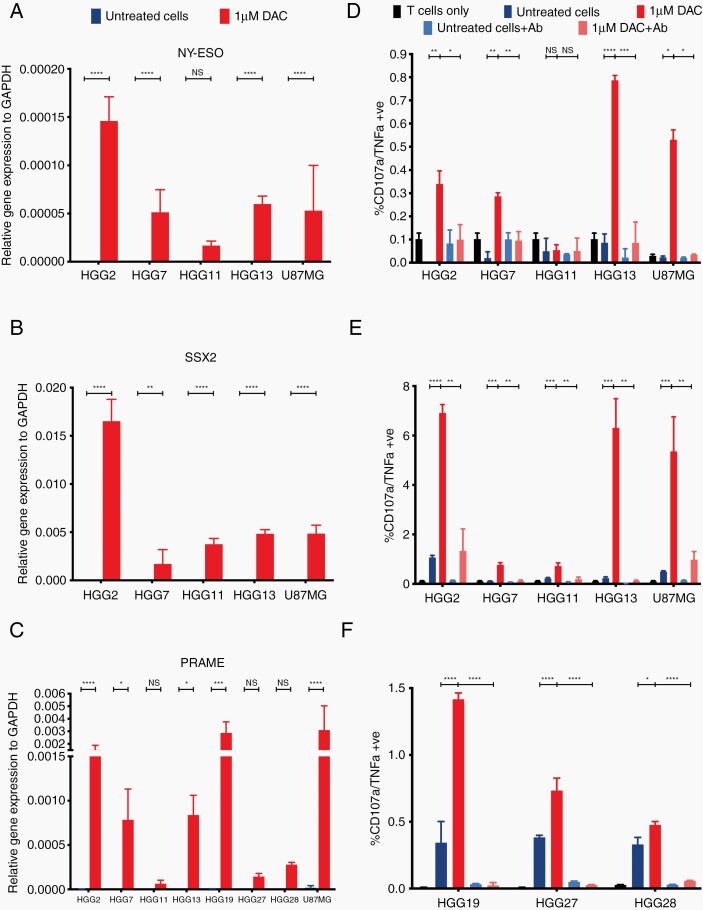

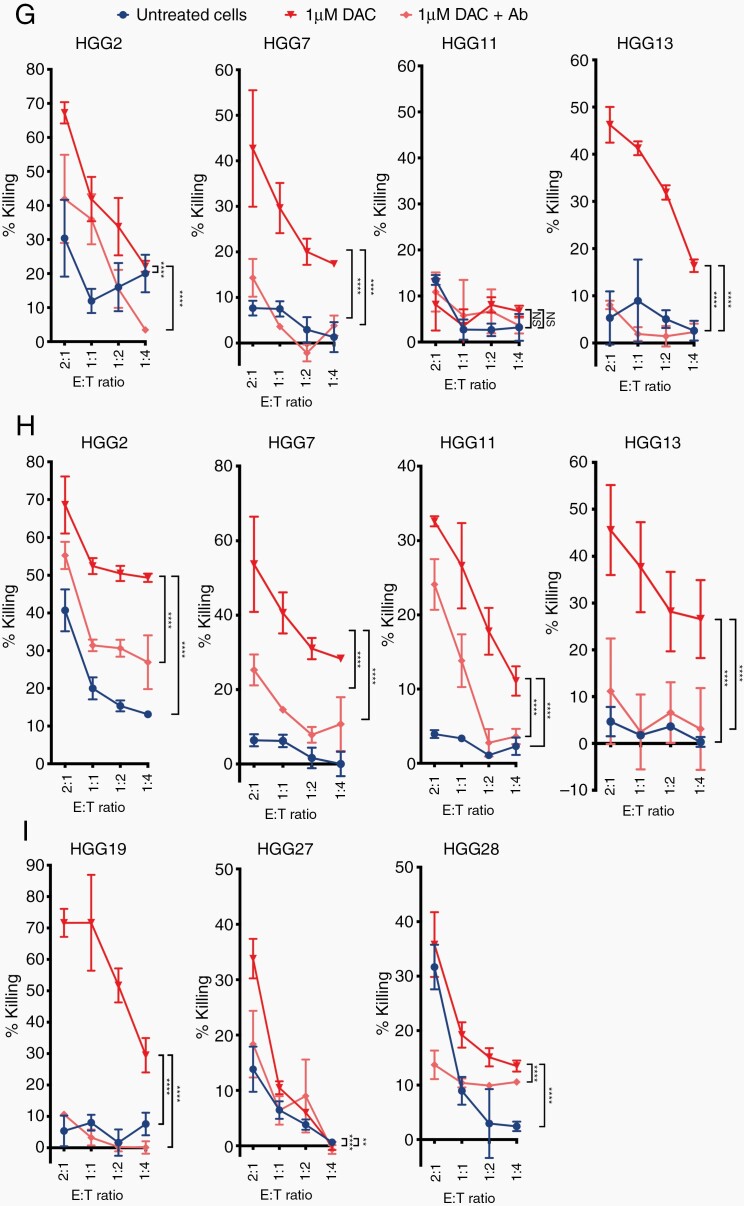
Increased expression of cancer testis antigens leads to increased CTL activation and killing in a TCR-MHC dependent fashion. Relative expression of (A) NY-ESO, (D) SSX2 and (G) PRAME to GAPDH as measured by RTqPCR. Expression is represented as 2^–∆∆CT^ and statistical significance as calculated by paired two-tailed student t test. Activation of (B) NY-ESO, (E) SSX2 and (H) PRAME specific CTL as measured by TNFα+/CD107a+ cells by flow cytometry. All cells pregated on CD3+/CD8+ population. (C) NY-ESO, (F) SSX2 and (I) PRAME specific CTL mediated killing as measured by LDH release against primary cell lines. Data shown are from biological triplicates representative of a minimum of 3 independent experiments. Ab—W6/32 MHC class I blocking antibody. Statistical significance as calculated by 2-way ANOVA. * *P <* .05, ** *P <* .01, *** *P <* .001, **** *P <* .0001.

To address whether increased expression of these potential neoantigens and CTA is sufficient to elicit an enhanced immune response, we generated neoantigen-specific T cells. Our initial approach was to stimulate autologous PBMC with the predicted HLA-A2-restricted neoantigens as previously described.^22^ Using this method, we were able to generate only one neoantigen-specific T-cell clone from the 4 patients tested, reflecting the poor capacity of GBM patients to generate neoantigen-specific responses and systemic immunosuppression from administration of dexamethasone. To generate additional peptide-specific T cells, we then performed *in-vitro* priming of healthy HLA-A2 donors. Using this method, we generated 34 unique T-cell clones reactive to 36 different peptides, which all showed dose-dependent activation against their specific peptides (Supplementary Figure 12). Of these, 5 T-cell clones were reactive against the relevant tumor lines, and 4 were specific for only the mutant peptide, making them suitable for further testing (Supplementary Figure 13, Figure 3).

### Increased Expression of Neoantigens and CTA Leads to Increased CTL Activation and Killing in a TCR:MHC Class I-Dependent Fashion

To interrogate the effects of DAC on neoantigen and CTA presentation by cancer cells we used antigen-specific T cells and measured their reactivity as a readout of antigen presentation. As we isolated T cells specific to 5 neoantigens (MYRF, MCM7, WDR91, LAMA3, and FAM122a) and had access to previously identified CTA-specific T cells against NY-ESO, SSX2, and PRAME, we decided to focus on those genes. Our PRAME-specific T cells were HLA-A3 restricted and hence, we used 3 additional GBM primary cell lines that were HLA-A3+ for functional experiments (HGG19/27/28; Supplementary Table 5). To quantify the change in expression of the genes encoding our neoantigens and CTA of interest we performed RT-qPCR. We found that NY-ESO, SSX2, and PRAME become re-expressed, to varying degrees, across U87MG and all the primary cell lines (Figure 3A–C) and this coincided with hypomethylation at the promoter site (Supplementary Figure 14). This upregulation in gene expression was sufficient to increase CTL activation (Figure 3D–F). This increase in activation was abrogated with the addition of an MHC class I-blocking antibody (W6/32; Figure 3D–F). Importantly, increased CTL activation led to greater CTL-mediated killing, which, again, was reduced with the addition of W6/32 (Figure 3G–I). Of note, there was no increase in expression of NY-ESO in HGG11, and consequently there was no increase in CTL activation and killing (Figure 3A,D,G).

**Fig. 4:**
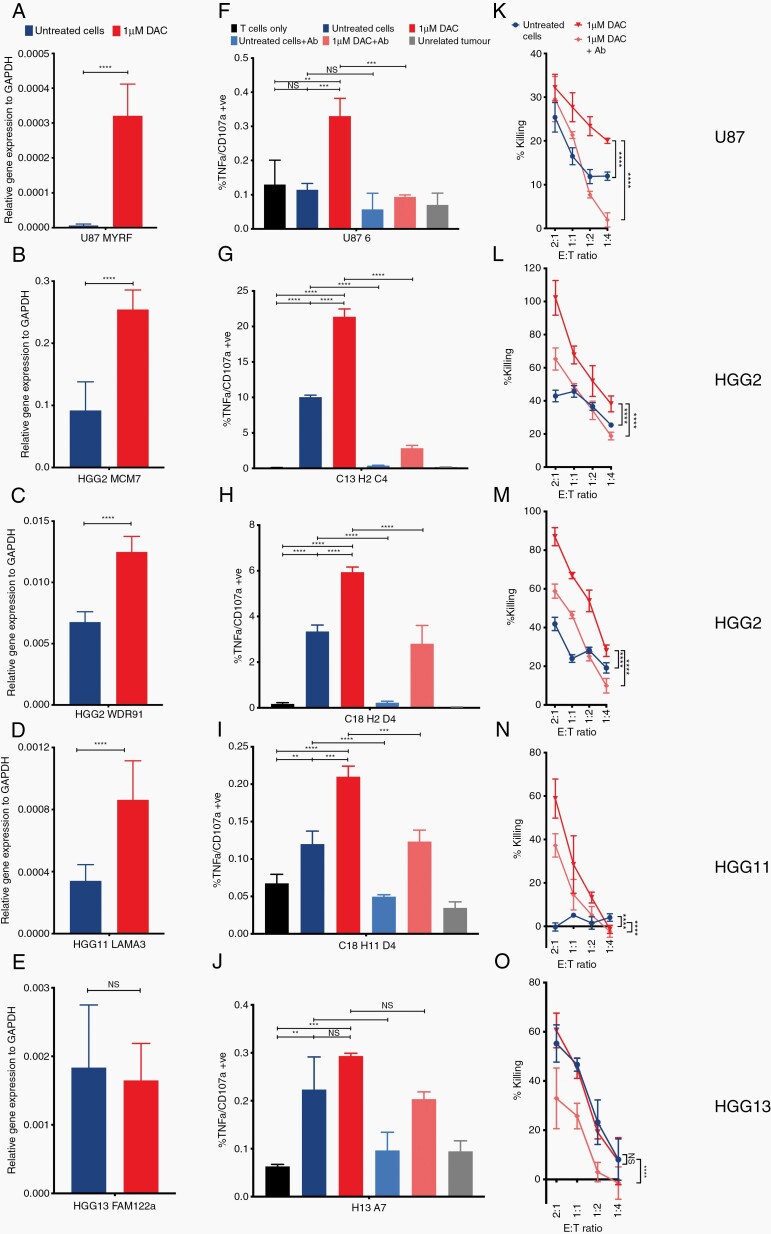
Increased expression of neoantigen leads to increased CTL activation and killing in a TCR-MHC dependent fashion. Bar plots showing relative gene expression of (A) MYRF, (D) MCM7, (G) WDR91, (J) LAMA3, (M) FAM122a to GAPDH as measured by RTqPCR for the relevant cell lines (A—U87MG; D+G—HGG2; J—HGG11, M—HGG13). Expression is represented as 2^–∆∆CT^ and statistical significance as calculated by paired two-tailed student t test. Activation of (B) MYRF, (E) MCM7, (H) WDR91, (K) LAMA3 and (N) FAM122a specific CTL as measured by TNFα+/CD107a+ cells by flow cytometry. All cells pregated on CD3+/CD8+ population. (C) MYRF, (F) MCM7, (I) WDR91, (L) LAMA3 and (O) FAM122a specific CTL mediated killing as measured by LDH release against primary cell lines. Data shown are from biological triplicates representative of a minimum of 3 independent experiments. Ab—W6/32 MHC class I blocking antibody. Statistical significance as calculated by 2-way ANOVA. * *P <* .05, ** *P <* .01, *** *P <* .001, **** *P <* .0001.

Four out of five neoantigens were significantly upregulated following treatment with DAC (Figure 4A–E). This was not always associated with hypomethylation at the promoter region, but interrogating the methylation array data, there was significant hypomethylation in other regions within the gene, including exons and other potential regulatory regions, such as enhancer regions (Supplementary Figure 15). Upregulation of the neoantigen led to increased CTL activation in a cell line-specific fashion (Figure 4F–J). This increase in CTL activation also led to increased killing, which could be blocked with W6/32 (Figure 4K–O). To ensure that this increase in CTL activation and killing was not due to a DAC-induced increase in MHC class I^34^, we show that there is a high level of MHC class I expression in all tumor lines at baseline and that expression remained unchanged following treatment with DAC (Supplementary Figure 16). In addition, for 1 neoantigen (FAM122a), there was no increase in expression following treatment with DAC (Figure 4E). Correspondingly, there was low-level CTL activation and killing when cocultured with untreated cells, which did not increase following treatment with DAC (Figure 4J,O). Altogether, these data indicate that DAC increases immunogenicity of GBM cancer cells, making them more susceptible to neoantigen and CTA-specific CTL-mediated killing. Furthermore, testing on the primary cell line HGG2, the duration of the effects of DAC treatment varied depending on the gene but persisted for a minimum of 2 weeks and up to greater than 4 weeks (Supplementary Figure 17).

### Decitabine Treatment of Tumor Cells Increases Activation of Patients’ Endogenous Tumor-Specific CTL

**Figure 5.**
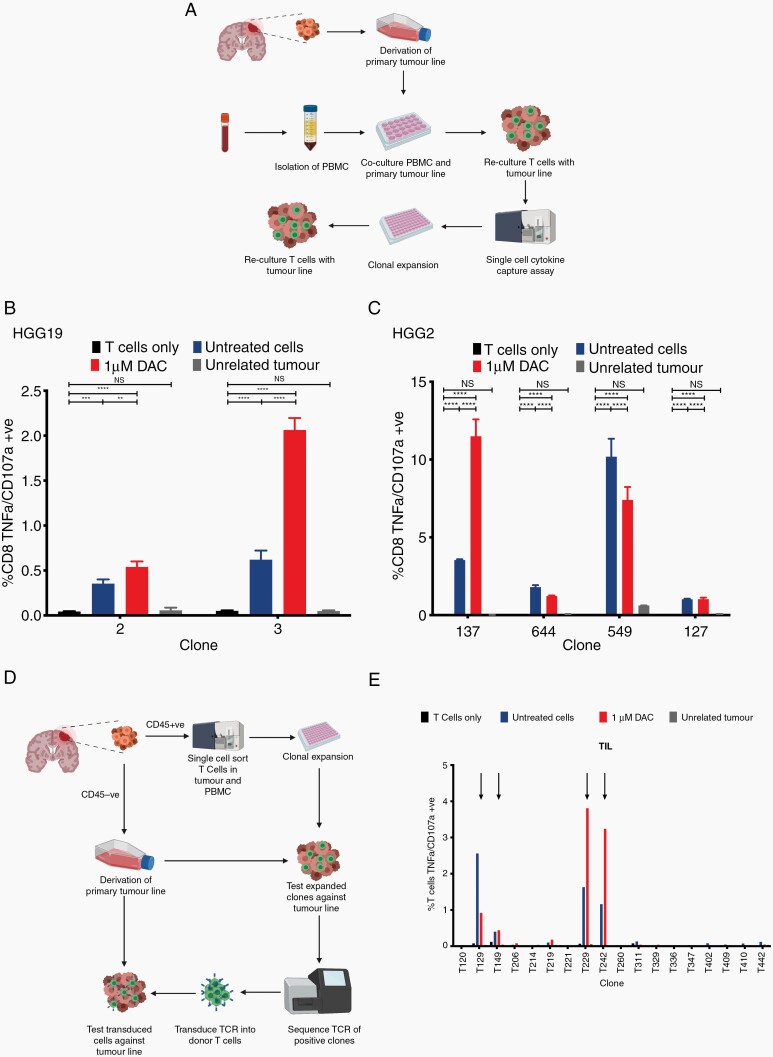
Some tumor reactive TCRs isolated from either MLTC and *ex-vivo* expansion also show increased reactivity against autologous primary GBM cell lines treated with DAC. (A) Schematic drawing of MLTC workflow. PBMC are cocultured with primary GBM cell lines through 2 rounds of stimulation. Potential reactive T cells are then isolated through cytokine capture assays and expanded. Expanded clones are then tested against autologous GBM cell line with/without DAC treatment as well as partially HLA-matched allogeneic GBM cell lines. (B,C) Bar plots showing activation of (B) HGG19 and (C) HGG2 specific T cells as measured by TNFα+/CD107a+ cells by flow cytometry. All cells pregated on CD3+/CD8+ population. (D) Schematic drawing of *ex-vivo* expansion workflow. T cells from PBMC/tumor were single cell sorted into 96 well plates and expanded *ex-vivo*. Expanded clones were tested against autologous GBM cell line with/without DAC treatment as well as partially HLA-matched allogeneic GBM cell lines. Selected tumor reactive T cells have their TCR sequenced and transduced into primary T cells for further testing. (E) Bar plot showing activation of primary T cells transduced with HGG37 specific TCRs as measured by TNFα+/CD107a+ cells by flow cytometry. Data shown are from biological triplicates representative of a minimum of 2 independent experiments. All cells pregated on CD3+/CD8+ population. Statistical significance calculated by 2-way ANOVA. * *P <* .05, ** *P <* .01, *** *P <* .001, **** *P <* .0001.

Finally, we addressed the question of whether endogenous CTL from patients would also show increased activation and targeting of DAC-treated autologous cell lines. To do this, we performed a mixed lymphocyte tumor coculture (MLTC) to isolate endogenous tumor-specific T cells (Figure 5A). We performed these experiments using PBMC from 3 patients where we were able to obtain adequate blood samples (HGG2, HGG13 and HGG19). Using this technique, we were able to isolate 29 tumor specific T-cell clones (11 CD8+, 18 CD4+; Supplementary Figure 18). We showed that they were able to recognize the tumor in a specific fashion, with no cross-reactivity against HLA-matched allogenic tumor lines. Additionally, focusing on the CD8+ T-cell clones that were cultured to sufficient numbers for further testing, we show that 3/6 tumor-reactive clones were more activated when cocultured with tumor cells treated with DAC compared to untreated cells (Figure 5).

Finally, we performed single-cell *ex-vivo* expansion of TIL and PBMC using freshly isolated samples from patient HGG37 (Figure 5D). Utilizing this method, we were able to generate 18/223 clones from the TIL and 26/314 from the PBMC. 5/18 TIL and 10/26 PBMC clones were tumor-reactive on an initial screening (Supplementary Figure 19). Upon further testing, 4/6 CD8+ T-cell clones that were able to be expanded in sufficient numbers showed increased reactivity to the DAC-treated autologous tumor cells compared to the untreated cells (Figure 5E).

## Discussion

Multiple proimmunogenic and antitumor effects have been associated with DAC. In preclinical models, DAC was shown to increase expression of antigen presentation machinery, interferon-stimulated genes and certain cancer testis antigens on tumor cells,^35–38^ including gliomas,^39–42^ as well as increasing the T-cell repertoire, reversing T-cell exhaustion,^43,44^ and improving the efficacy of immunotherapies (CAR T cells^45^ and anti-PD-1^46^). In the clinic, DAC was licensed for the treatment of myelodysplastic disease^47^ and is used off-label for high risk acute myeloid leukemia,^48^ due to its potent myelosuppressive effect. DAC was also shown to reverse locus-specific DNA hypermethylation inhibiting growth, invasion, and migration of cancer cells.^49^

In this study, we describe a novel mechanism that mediates the immunogenic effects of DAC. We show that it increases the expression of neoantigens *in-vitro*. Whilst there have been reports of specific CTA, such as NY-ESO, being upregulated by DAC in GBM,^39–41,49^ to our best knowledge, we are also the first to report a global upregulation of CTA in GBM. Promoter methylation was reported as the primary mechanism regulating the expression of CTA^50^ which makes them ideal targets for hypomethylating therapies. On the other hand, neoantigens are generated from random mutations in the genome and their silencing mechanisms seem to be more variable. This notwithstanding, hypermethylation of genes harboring neoantigens was already identified as an immune escape mechanism in lung cancer patients.^51^ Here, we formally demonstrate that reversing DNA hypomethylation using DAC increases the susceptibility of cancer cells to neoantigen- and CTA-specific T-cell killing, in a TCR:MHC-I dependent manner.

Recently, Leko et al. have identified neoantigen-reactive CD4+ T cells specific to one mutation carried by one GBM patient,^52^ out of five patients studied. Similarly, in the current study using a cohort of four patients, we isolated one neoantigen-specific CD8+ T-cell clone from the peripheral blood of one GBM patient. Taken together, these two pioneer studies are demonstrating the low number of endogenous neoantigen-specific T cells in GBM patients, which reflects the immunosuppressive microenvironment and low TMB of GBM and possibly the systemic immunosuppression caused by dexamethasone (a strong corticosteroid) treatment. Importantly, we observed that isolation of neoantigen-reactive CTL is more efficient when priming PBMC from healthy donors which can be a better alternative for poorly immunogenic cancers. It remains to be known whether antigen-specific CD4+ T cells—a dominant subset in neoantigen-specific immune responses^15,16^—can also be isolated using this method.

Immunotherapies, namely checkpoint inhibition and cancer vaccines, have been showing poor efficacy in GBM.^13–16^ Despite these disappointing results, GBM patients with high TMB caused by germline mismatch repair deficiency and consequent high neoantigen load, had durable responses to checkpoint inhibitor therapy.^53,54^ We hypothesize that by increasing the expression of neoantigens and CTA in GBM, DAC can provide the immunostimulatory drive to overcome resistance to immunotherapies in poorly immunogenic cancers. Indeed, ongoing clinical trials are testing the combination of DAC with immune checkpoint inhibitors in multiple solid and blood malignancies (NCT03019003, NCT03066648, NCT03969446, NCT02608268), namely in anti-PD-1-refractory tumors (NCT04611711). Similar clinical trials in GBM patients are of utmost importance to understand if DAC is capable of increasing the immunogenicity of GBM *in-vivo* and whether that is sufficient to reactivate already existing or stimulate de novo T-cell responses *in -vivo*.

In conclusion, we demonstrate that DAC can improve the immunogenicity of GBM, by increasing neoantigen and CTA expression *in-vitro*. This provides a rationale to study new treatment combinations to overcome the barriers to successful implementation of existing immunotherapies such as checkpoint inhibition and vaccination therapies. We have also shown that it is possible to isolate multiple tumor-specific T cells from GBM patients and healthy donors which show increased reactivity against GBM cells when treated with DAC. This can open another potential therapeutic avenue—the combination of DAC therapy with adoptive cell transfer of *ex-vivo*-expanded tumor-specific T cells.

## Supplementary Material

noac107_suppl_Supplementary_Figures

noac107_suppl_Supplementary_File

noac107_suppl_Supplementary_Material

noac107_suppl_Supplementary_Tables
