# Supplementary material for: Decitabine increases neoantigen and cancer testis antigen expression to enhance T-cell–mediated toxicity against glioblastoma
Source: Neuro Oncol. 2022 Apr 25;24(12):2093–106. doi: 10.1093/neuonc/noac107 (PMC9713507; doi:10.1093/neuonc/noac107)
Supplement: noac107_suppl_Supplementary_Figures [file noac107_suppl_supplementary_figures.pdf]

Supplementary figures

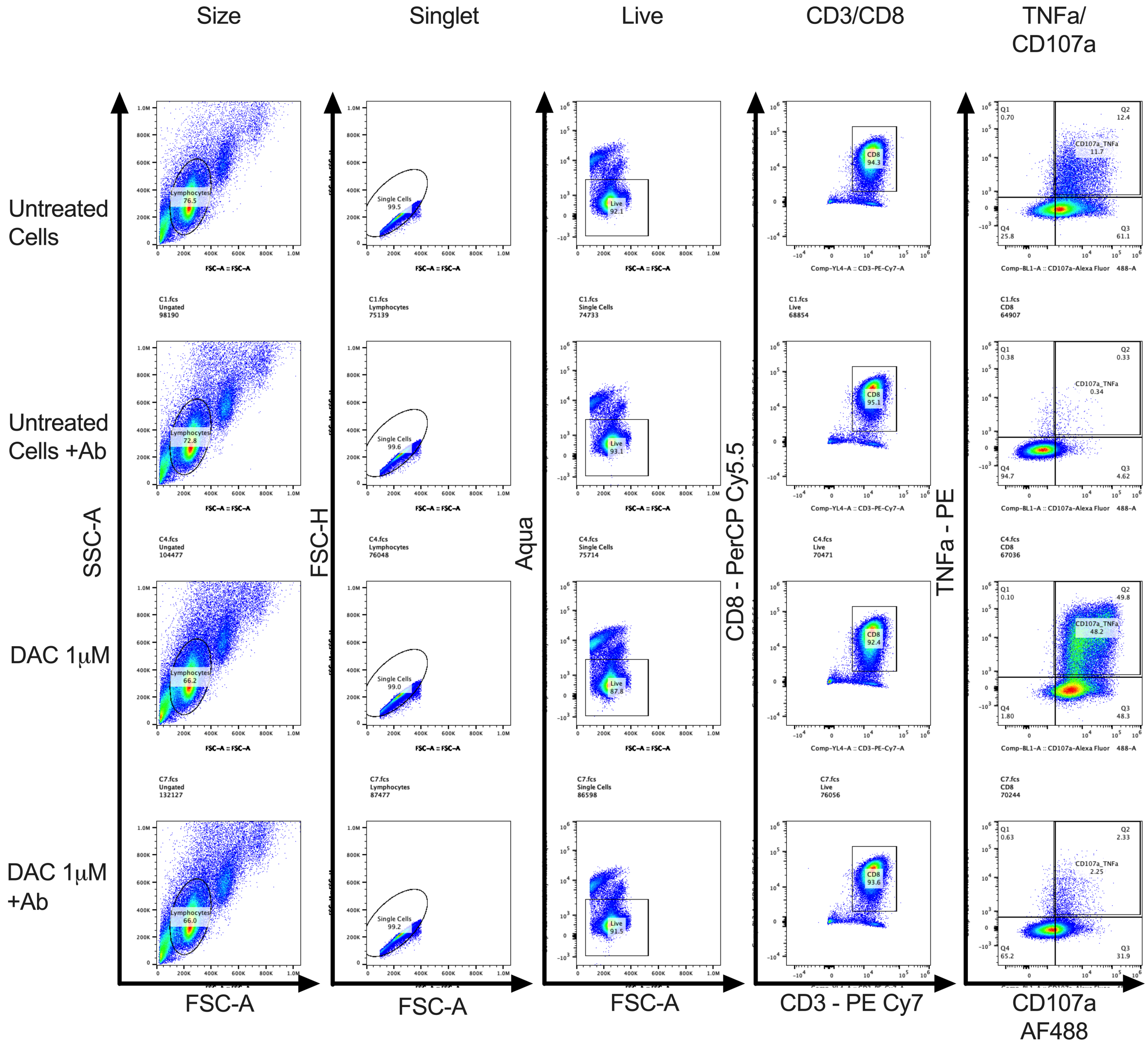

Supplementary figure 1. Example gating strategy for T cell activation assay. Dot plots illustrating gating strategy for T cell activation assay. Cells are gated by size>singlet>live>CD3+/CD8+ populations.

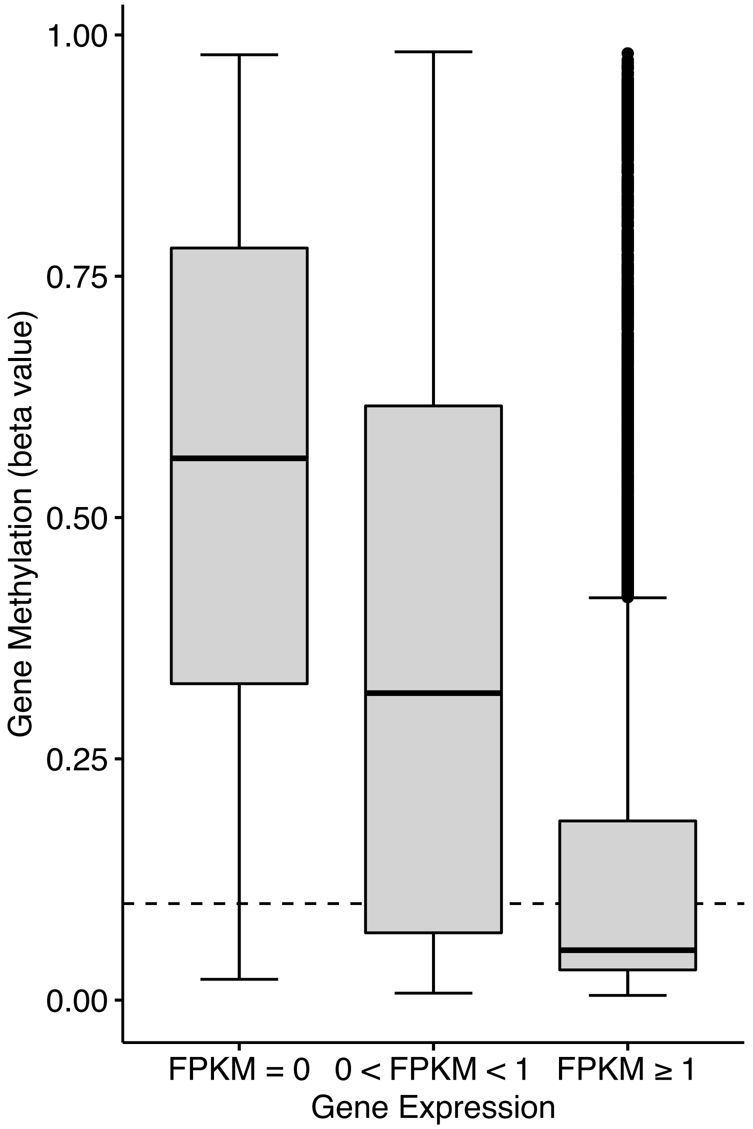

Supplementary figure 2. Unexpressed/lowly expressed mutations have higher mean methylation than expressed mutations. Box and whisker plot showing methylation of genes harbouring non-silent mutations from the TCGA based upon their expression levels.

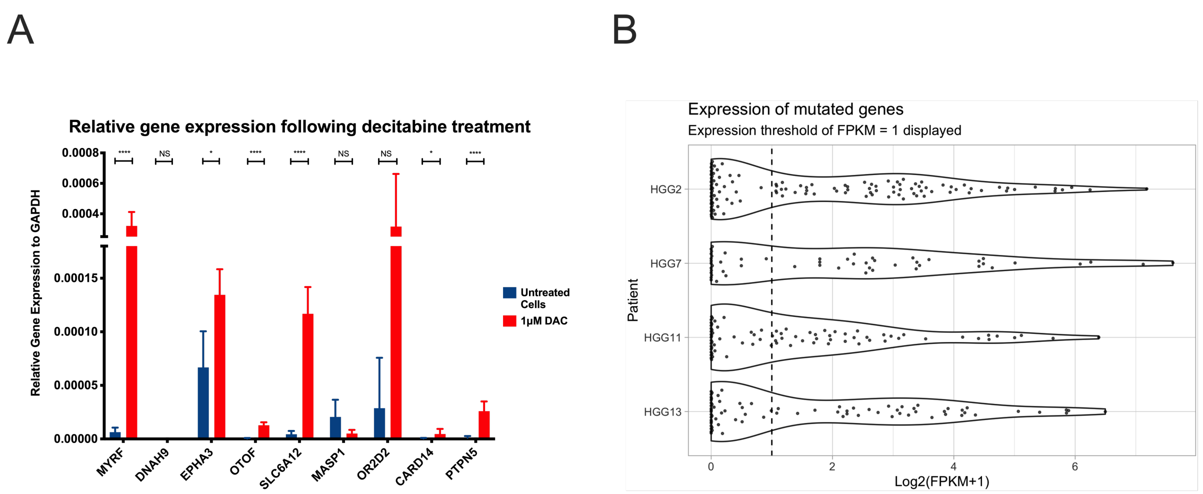

Supplementary figure 3. The majority of potential neoantigen encoding mutations are not/lowly expressed. (A) Bar chart showing relative expression of genes with potential neoantigen encoding mutations to GAPDH as measured by RTqPCR. Data generated for U87MG cell line. Expression is represented as $2^{-\Delta\Delta CT}$ and statistical significance as calculated by paired two-tailed student t test. (B) Violin plot showing FKPM values of genes with potential neoantigen encoding mutations in primary cell lines not treated with DAC. Data generated from primary cell lines. Data shown in (A) are from technical triplicates representative of 2 biological replicates. Data shown in (B) are from biological triplicates. Statistical significance calculated by paired 2-tailed student t-test. * $p \leq 0.05$, ** $p \leq 0.01$, *** $p \leq 0.001$, **** $p \leq 0.0001$

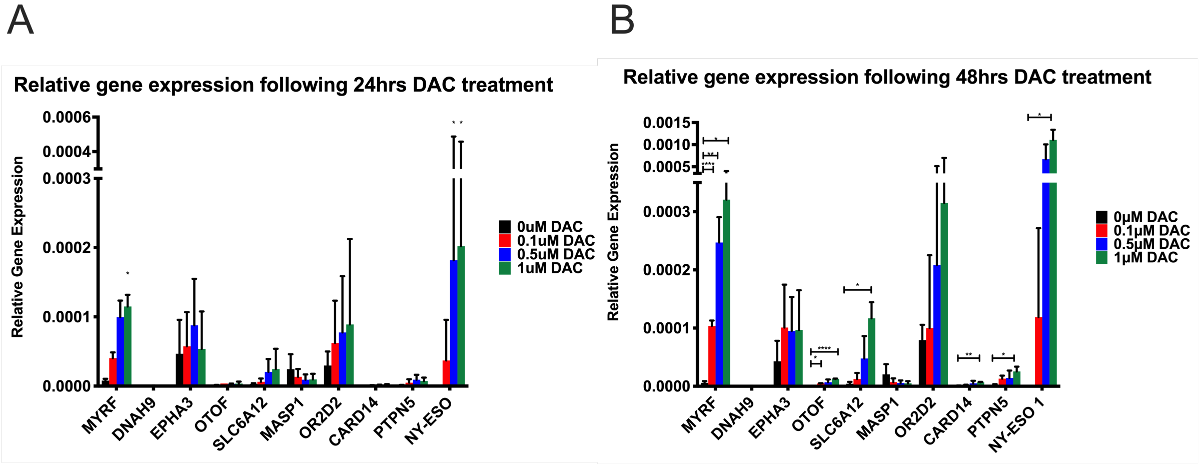

Supplementary figure 4. DAC treatment shows a response curve both based on concentration and treatment duration. Bar charts showing relative expression of genes following treatment with different concentration of DAC for (A) 24 and (B) 48 hours with potential neoantigen encoding mutations to GAPDH as measured by RTqPCR. Data generated for U87MG cell line. Expression is represented as $2^{-\Delta\Delta CT}$ and statistical significance as calculated by paired two-tailed student t test. Data is shown for biological duplicates.

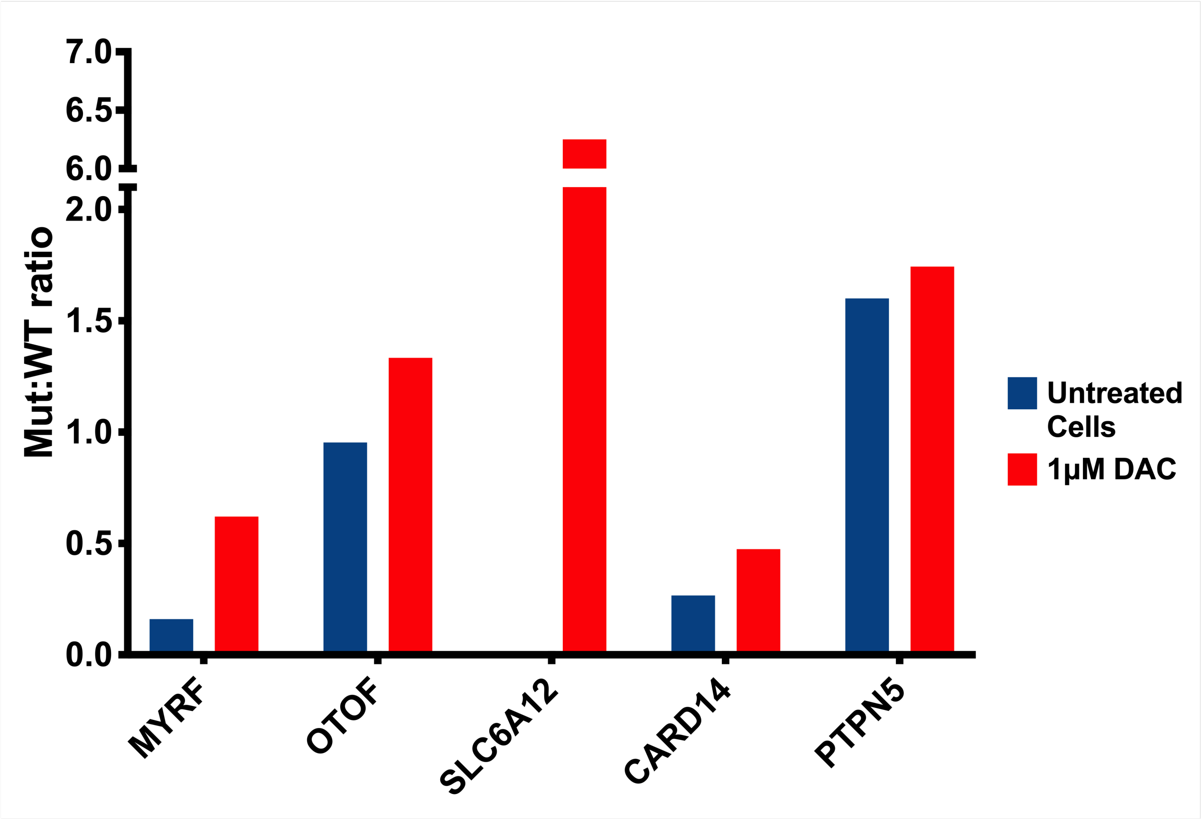

Supplementary figure 5. Mutant:wild-type ratios are not changed or increased following treatment with DAC. Bar charts representing the mutant:wild-type allele ratio following treatment with DAC. Ratio expressed based on number of reads for mutant vs. wild-type allele on targeted NGS.

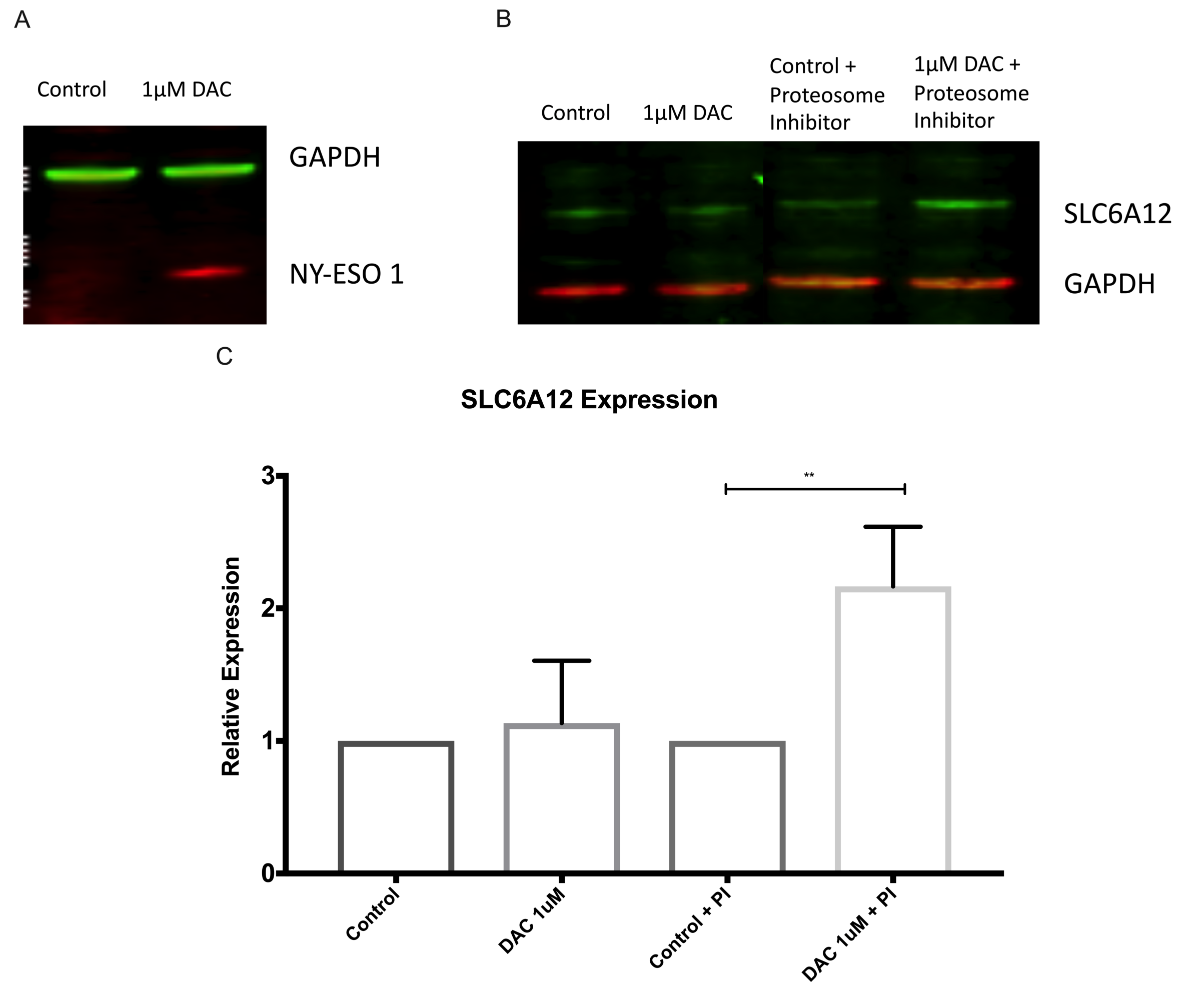

Supplementary figure 6. DAC treatment increases protein expression of both CTA and neoantigens. Representative Western blots showing expression of NY-ESO (A) and potential neoantigen harbouring protein, SLC6A12 (B). (C) Bar plot showing quantification of fluorescence seen in (B). Data representative of 3 biological replicates. Significance as determined by 2-way ANOVA. * $p \leq 0.05$, ** $p \leq 0.01$, *** $p \leq 0.001$, **** $p \leq 0.0001$

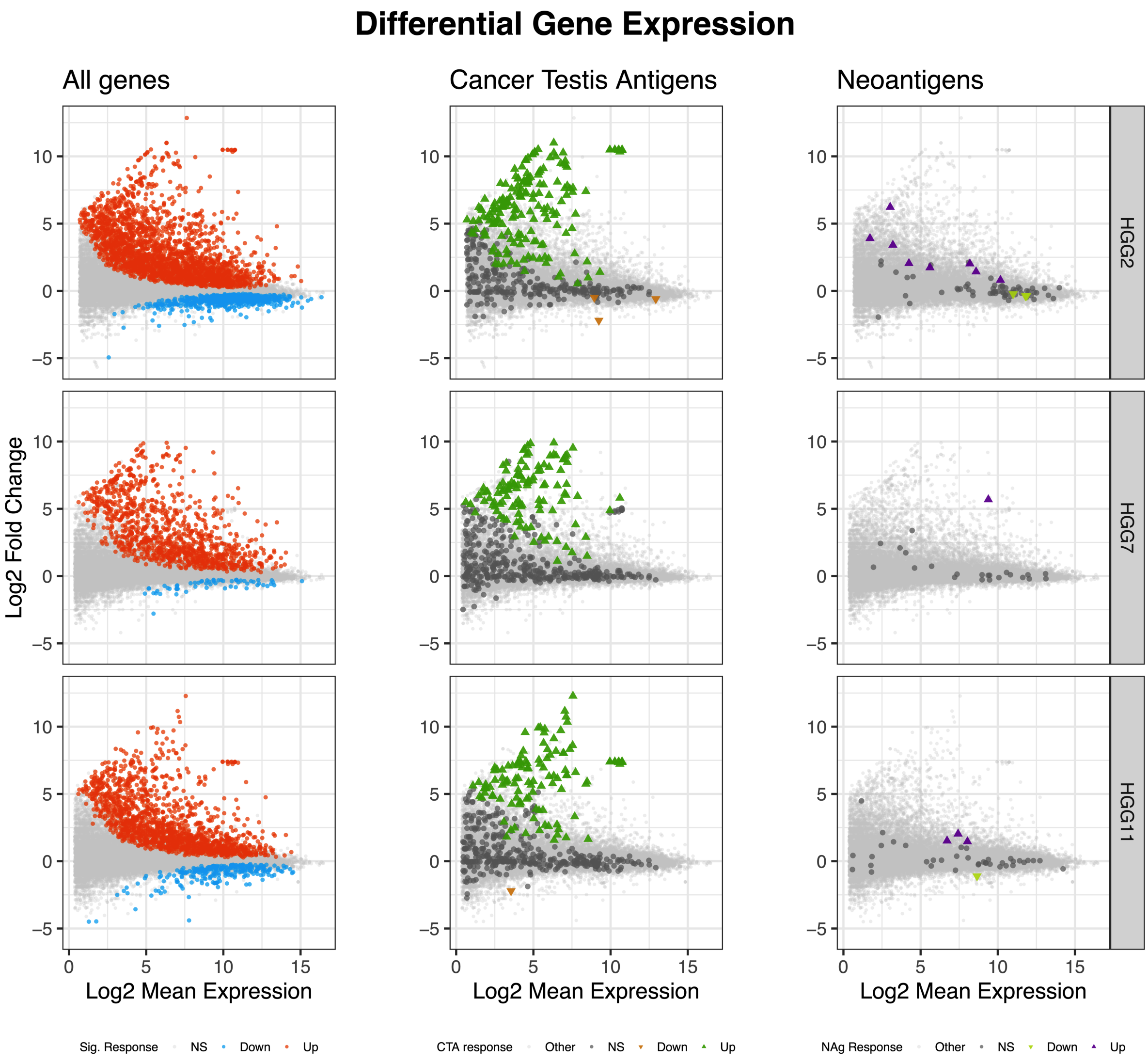

Supplementary figure 7. **Decitabine increases expression of both cancer testis antigens and neoantigens in primary GBM cell lines.** MA plots comparing expression of all genes, cancer testis antigens and neoantigens in primary GBM cell lines with and without DAC treatment for HGG2, HGG7 and HGG11. Coloured dots represent individual genes with significantly ($p \leq 0.05$) altered expression following DAC treatment, direction of arrow signifies direction of change. Data are from biological triplicates.

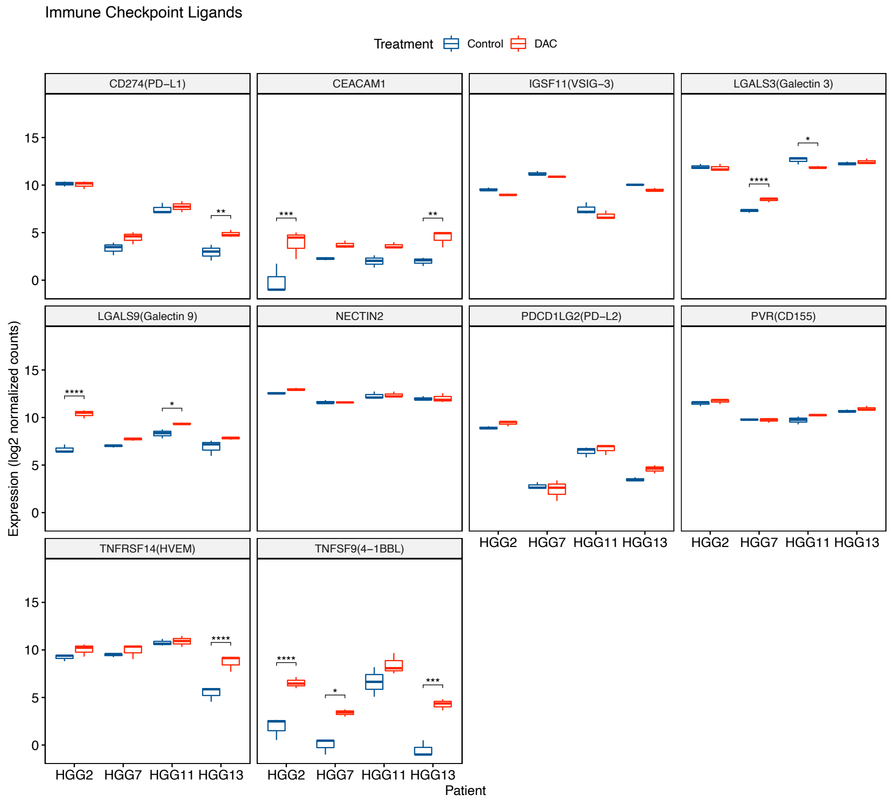

Supplementary figure 8. Primary tumour cell lines show variable baseline expression of immune checkpoint ligands and exhibit variable changes in expression following DAC treatment. Box and whisker plots of normalized expression of immune checkpoint ligands of primary cell lines with/without treatment with DAC as determined by RNAseq. Data are from biological triplicates. Significance as determined by DESeq * $p \leq 0.05$, ** $p \leq 0.01$, ***$p \leq 0.001$, **** $p \leq 0.0001$

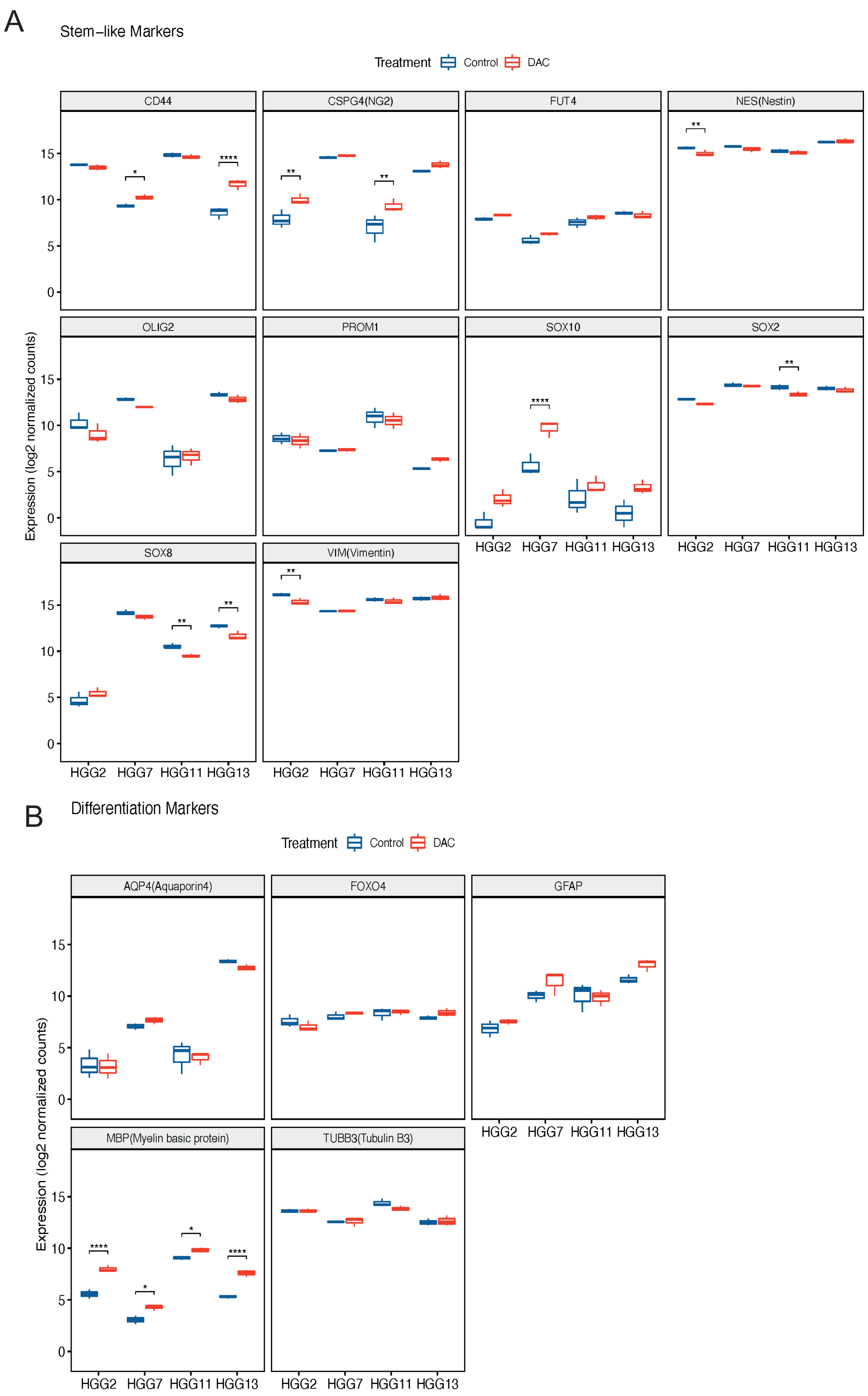

Supplementary figure 9. Primary tumour cell lines broadly maintain their phenotype following DAC treatment. Box and whisker plots of normalized expression of (A) stem-like and (B) differentiation markers of primary cell lines with/without treatment with DAC as determined by RNAseq. Data are from biological triplicates. Significance as determined by DESeq * p≤0.05, ** p≤0.01, ***p≤0.001, **** p<u><</u>0.0001

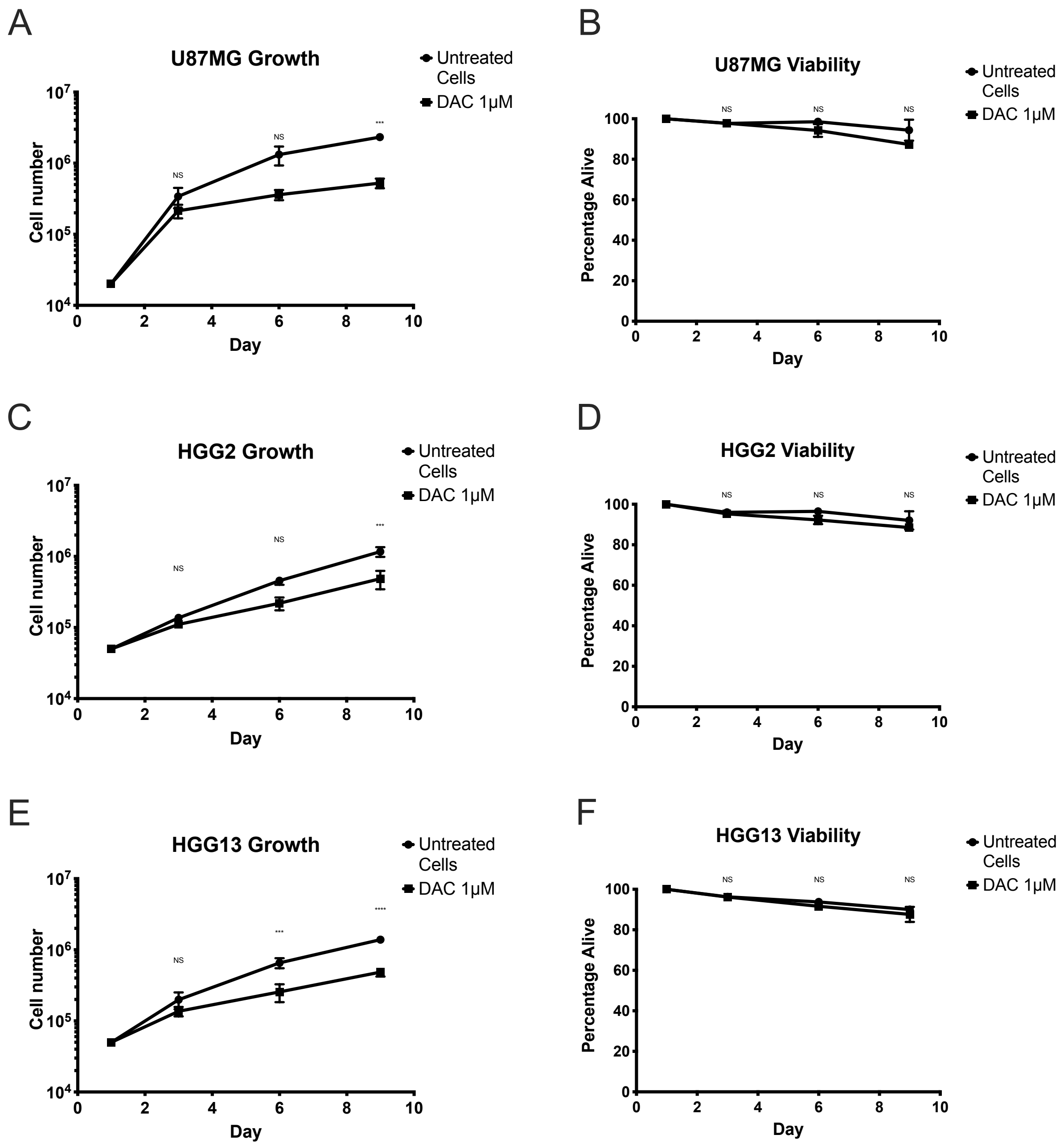

Supplementary Figure 10. Decitabine treatment reduces cell proliferation but not cell viability. (A, C, E) Growth and (B, D, F) viability as measured by trypan blue staining. Data shown are from technical triplicates representative of 2 biological replicates. Significance as determined by 2-way ANOVA. * p≤0.05, ** p≤0.01, ***p≤0.001, **** p≤0.0001

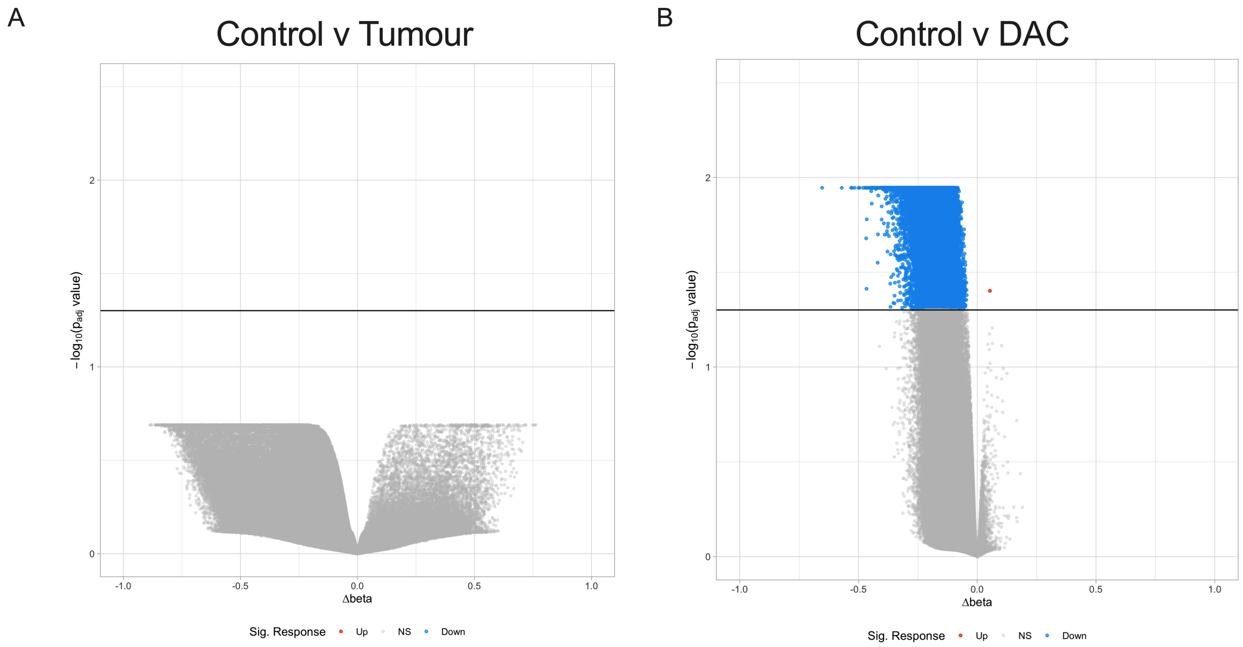

Supplementary figure 11. Similar methylation pattern of *ex vivo* tumour samples compared to the patient or primary cell line and DAC-driven global hypomethylation of the genome. Volcano plot showing changes in methylation of individual probes between (A) control (primary cell lines) v tumour samples (*ex vivo* samples) and (B) control (primary cell lines) v DAC treated cell lines. Coloured dots represent individual genes with significantly ($p \leq 0.05$) altered expression following DAC treatment, direction of arrow signifies direction of change. Tumour – primary tumour sample; control – primary tumour cell lines not treated with DAC; DAC – primary tumour cell lines treated with DAC. Data are from biological duplicates.

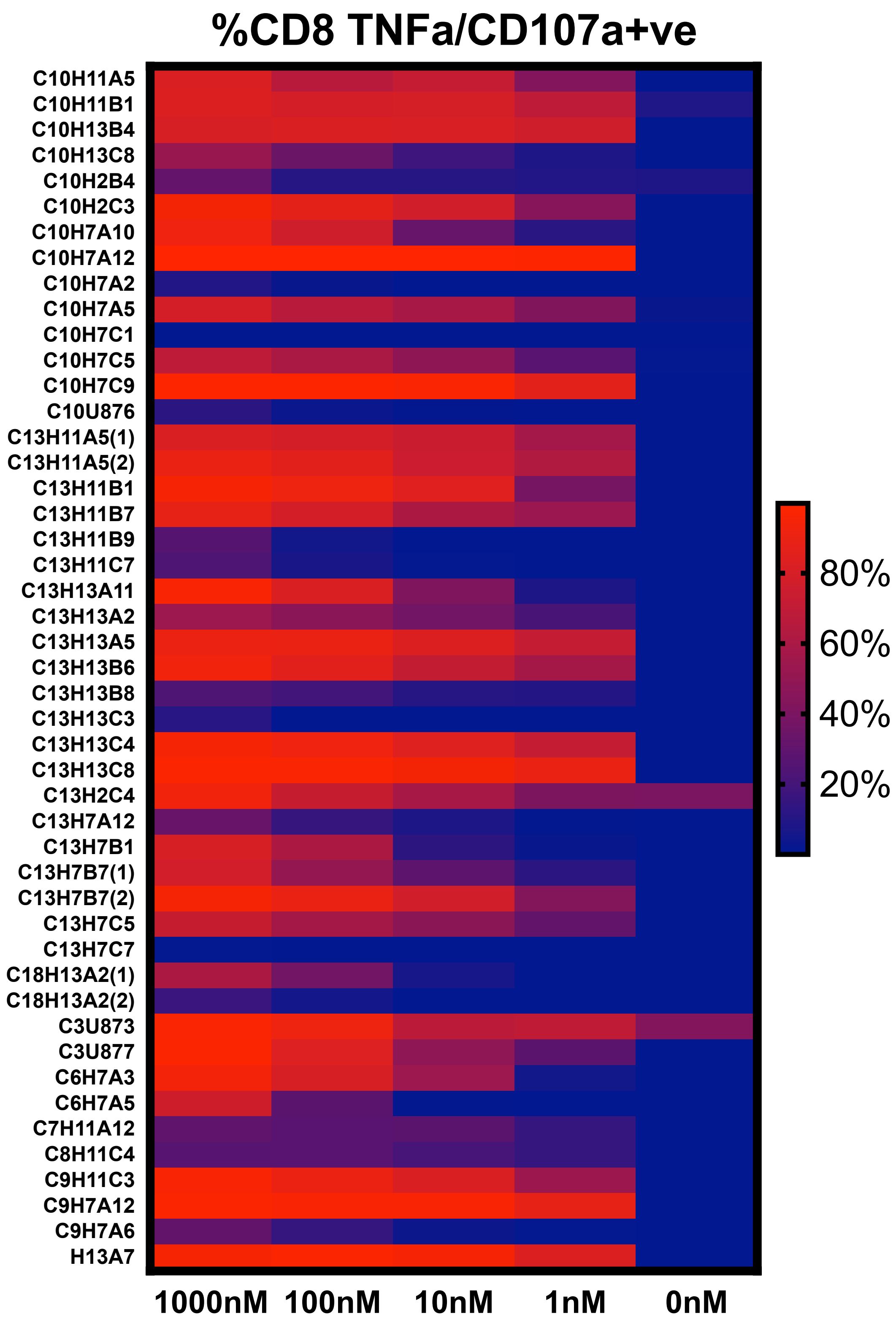

Supplementary figure 12. Peptide specific T cells isolated show reactivity against the peptide in a concentration dependent manner. Heat map showing response to the relevant peptide, at concentrations ranging from 0-1000nM, for all T cell clones isolated. Response is measured as % cells TNFα+/CD107a+ and depicted in different shades of grey, with darker grey meaning higher responses. Cells are pre-gated on CD3+/CD8+ve. Data shown is from 3 biological replicates each performed as technical duplicates.

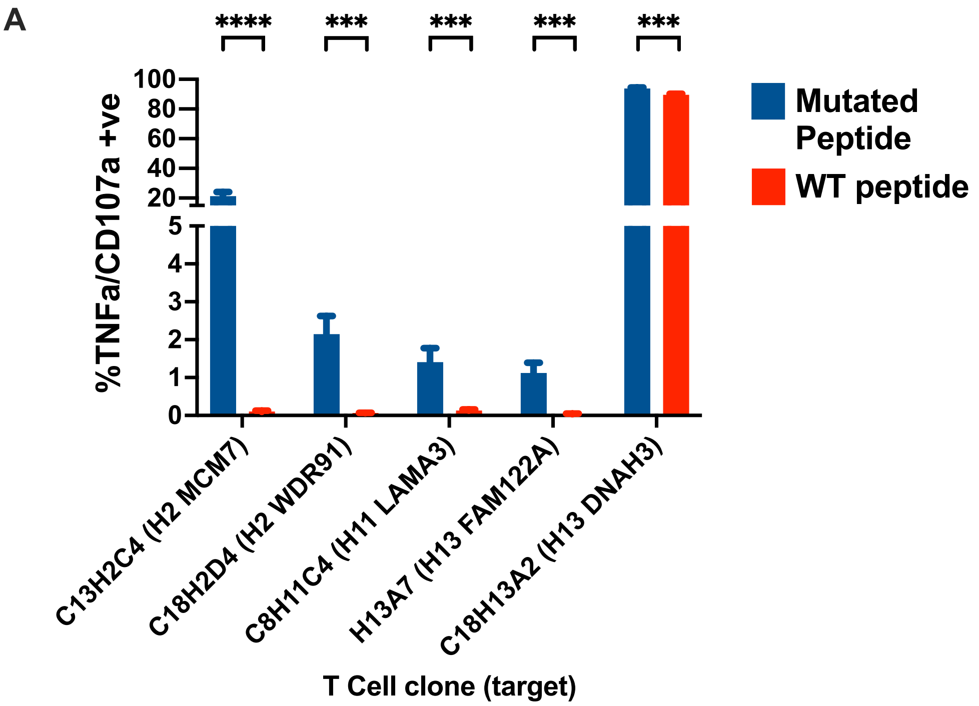

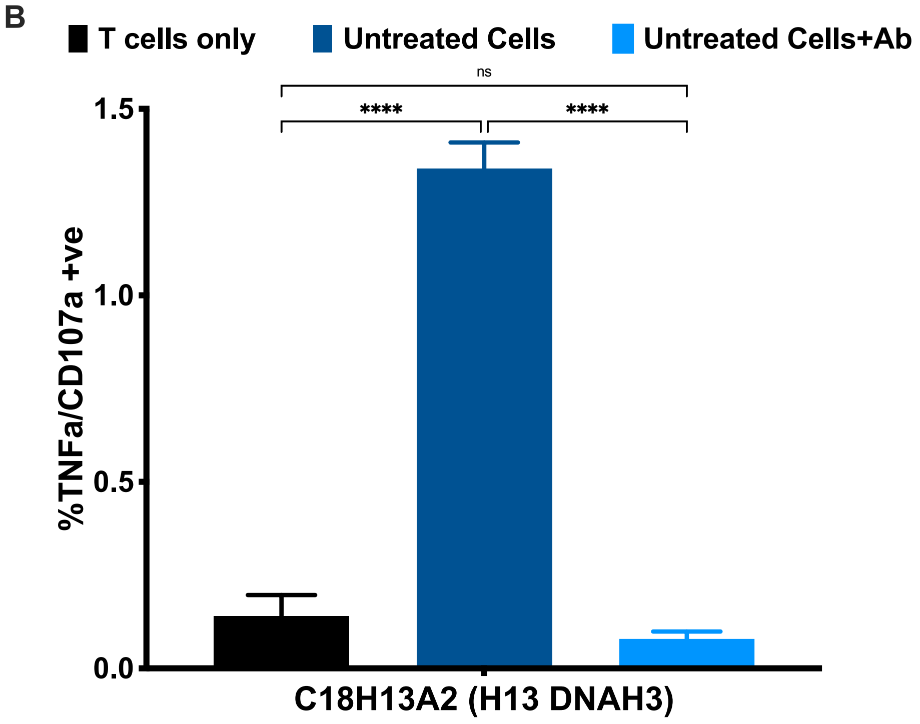

Supplementary figure 13. 4/5 peptide specific T cells were specific to the mutant peptide only. (A) Bar chart showing activation of T cells following co-culture with HLA-A2+ cells pulsed with 1uM peptide. (B) activation of C18H13A2 T cells following co-culture with HGG13 cells in the presence or absence of W6/32. Activation of T cells as measured by TNFα+/CD107a+ cells by flow cytometry. All cells pre-gated on CD3+/CD8+. Data shown from 3 biological replicates. Significance as determined by (A) paired 2-tailed student t-test, (B) One-way Anova. * p≤0.05, ** p≤0.01, ***p≤0.001, **** p≤0.0001

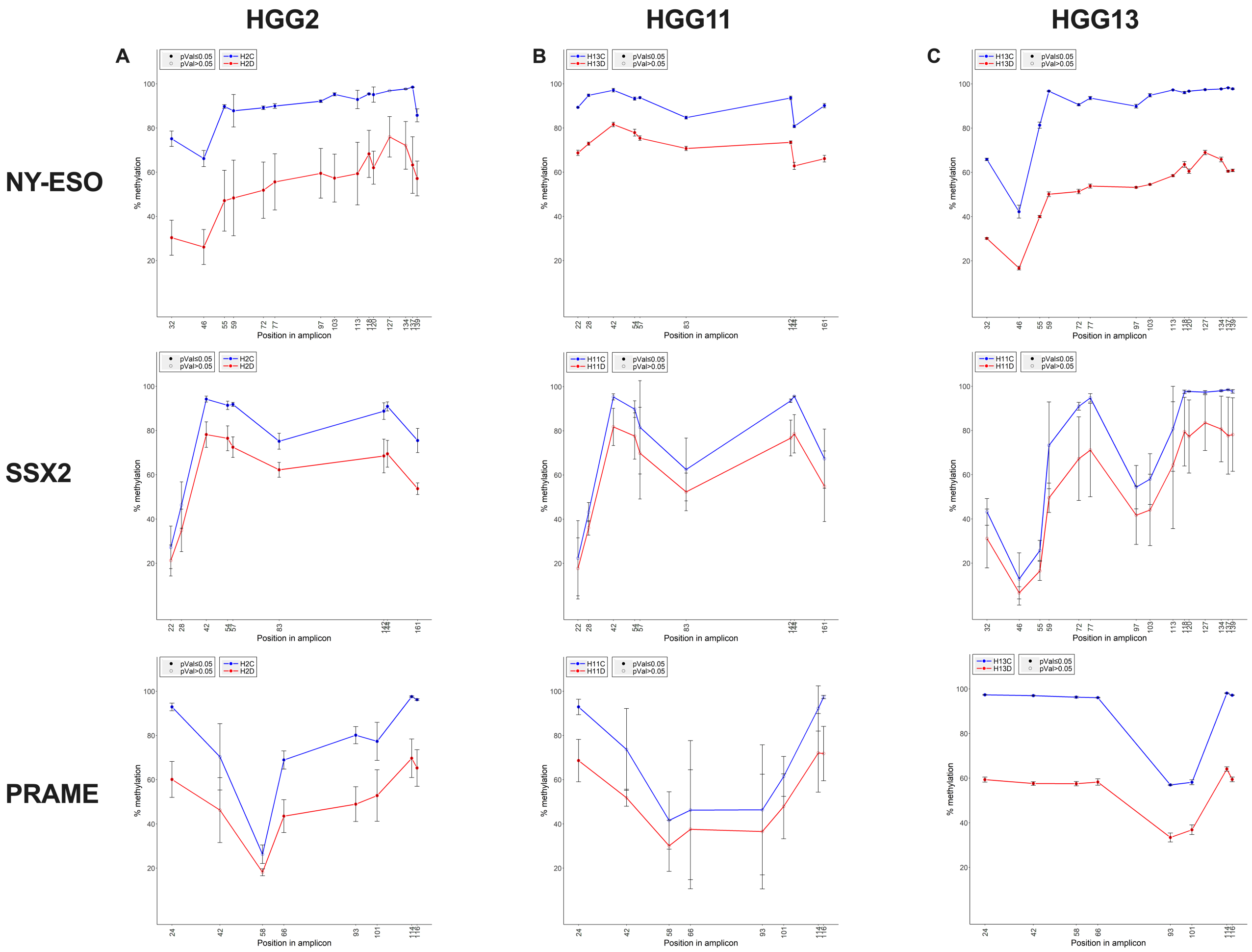

Supplementary figure 14. DAC treatment leads to significant hypomethylation in the promoter region of CTA. Chart showing results of targeting bisulphite sequencing in the promoter region of (A) NY-ESO and (B) SSX2 and (C) PRAME. Results are mean of 3 biological replicates. Statistical significance as calculated by 2-way ANOVA. Solid dots represent significant (p<u><</u>0.05) difference between DAC and control methylation.

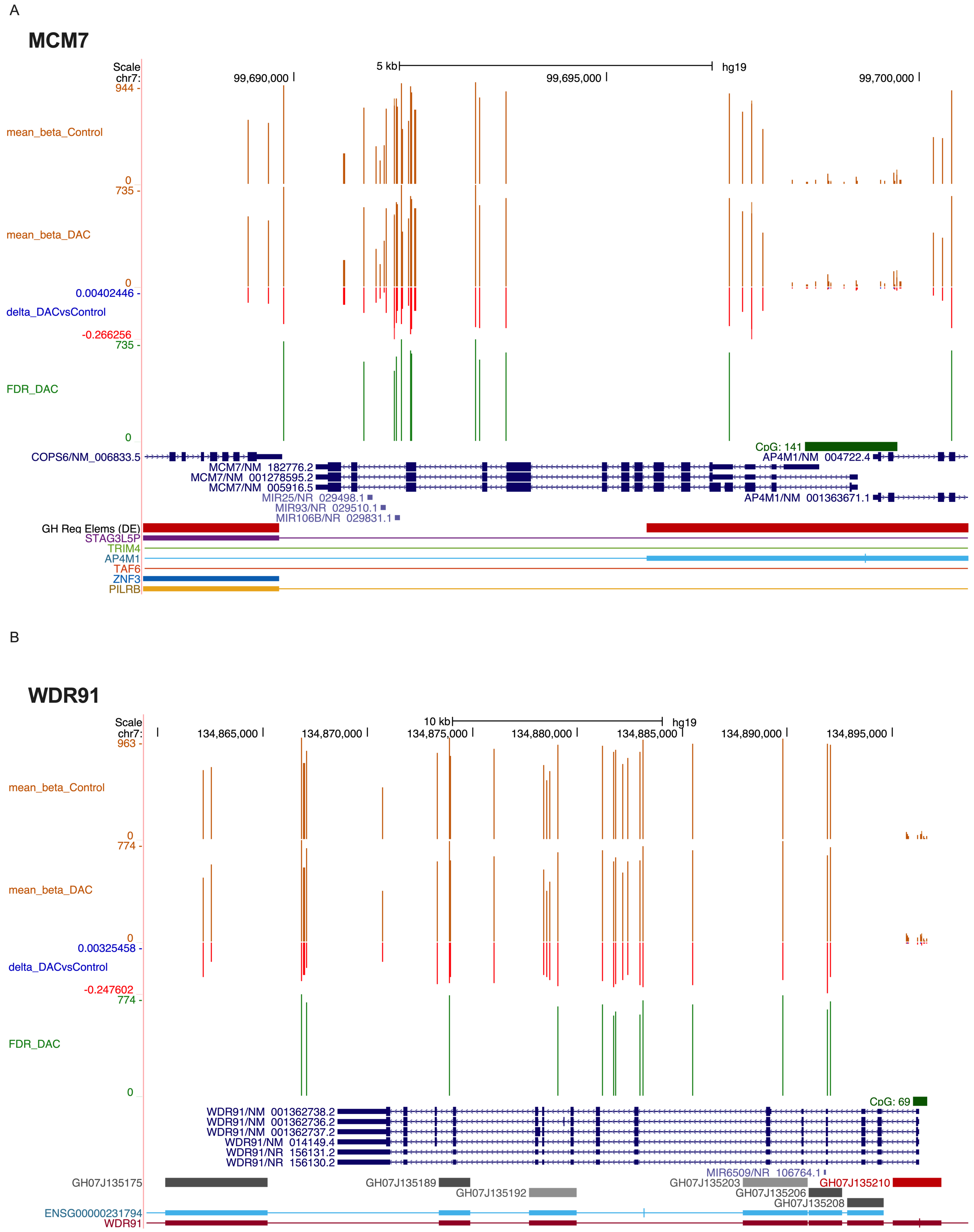
A
MCM7
Scale
chr7:
5 kb
hg19
99,690,000
99,695,000
99,700,000
944 -
mean_beta_Control
0
735 -
mean_beta_DAC
0
0.00402446 -
delta_DACvsControl
-0.266256
735 -
FDR_DAC
0
CpG: 141
COPS6/NM_006833.5
AP4M1/NM_004722.4
MCM7/NM_182776.2
MCM7/NM_001278595.2
MCM7/NM_005916.5
MIR25/NR_029498.1
AP4M1/NM_001363671.1
MIR93/NR_029510.1
MIR106B/NR_029831.1
GH Reg Elems (DE)
STAG3L5P
TRIM4
AP4M1
TAF6
ZNF3
PILRB
B
WDR91
Scale
chr7:
10 kb
hg19
134,865,000
134,870,000
134,875,000
134,880,000
134,885,000
134,890,000
134,895,000
963 -
mean_beta_Control
0
774 -
mean_beta_DAC
0
0.00325458 -
delta_DACvsControl
-0.247602
774 -
FDR_DAC
0
CpG: 69
WDR91/NM_001362738.2
WDR91/NM_001362736.2
WDR91/NM_001362737.2
WDR91/NM_014149.4
WDR91/NR_156131.2
WDR91/NR_156130.2
MIR6509/NR_106764.1
GH07J135175
GH07J135189
GH07J135192
GH07J135203
GH07J135210
GH07J135206
GH07J135208
ENSG00000231794
WDR91

C

LAMA3

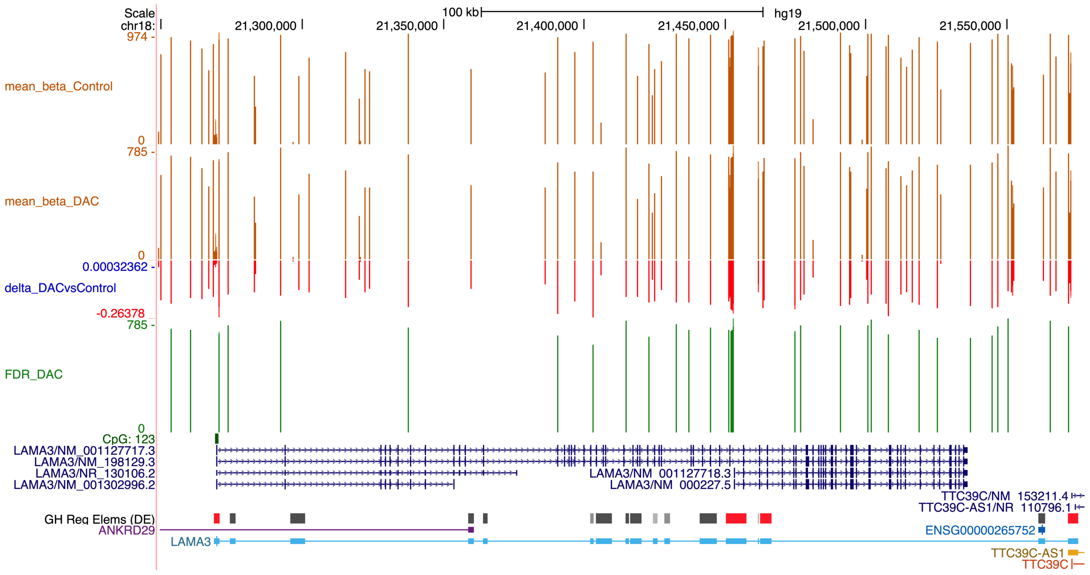

D

FAM122a

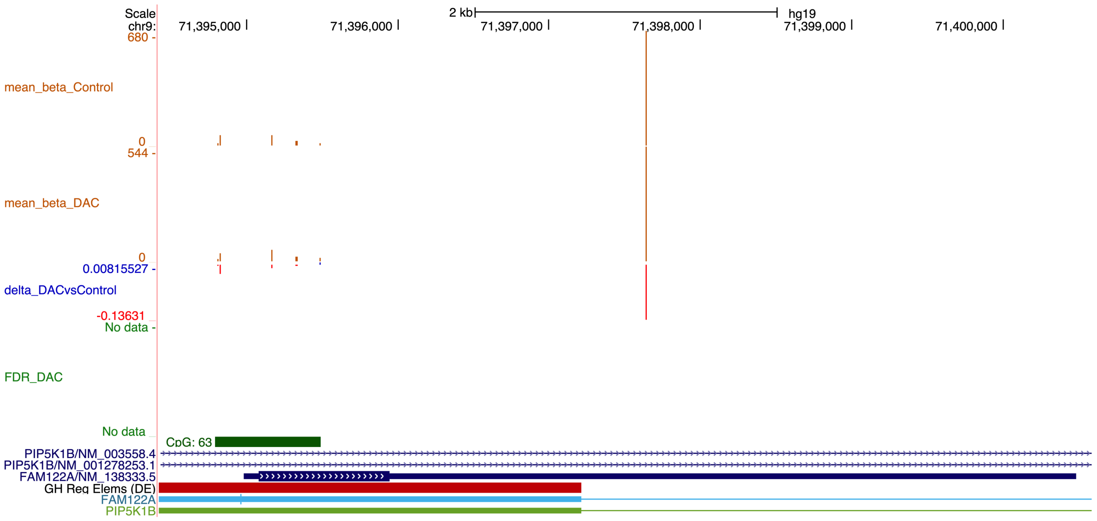

Supplementary figure 15. DAC treatment of cell lines leads to hypomethylation of genes harbouring potential neoantigen encoding mutations. Charts showing change in methylation of CpG sites as obtained from Infinium EPIC array of (A) MCM, (B) WDR91 and (C) LAMA3 and (D) FAM122a. Mean beta control/DAC, absolute methylation track reflecting array output going from 1, no methylation, to 1,000, fully methylated. delta DACvsControl, a differential methylation track showing proportional change going from −1.0 (100% loss, red) to +1.0 (100% gain, blue). FDR DAC, a significance score track showing only those sites whose differential methylation meets the cut-off criterion of false discovery rate<0.05. Blue track below represents RefSeq track; multicoloured track below represents GeneHancer Regulatory elements and gene interactions track. Red bars represent promoter regions, grey bars represent enhancer regions, multicoloured bars are visual representations of enhancer-gene interactions. Data are from 3 biological replicates.

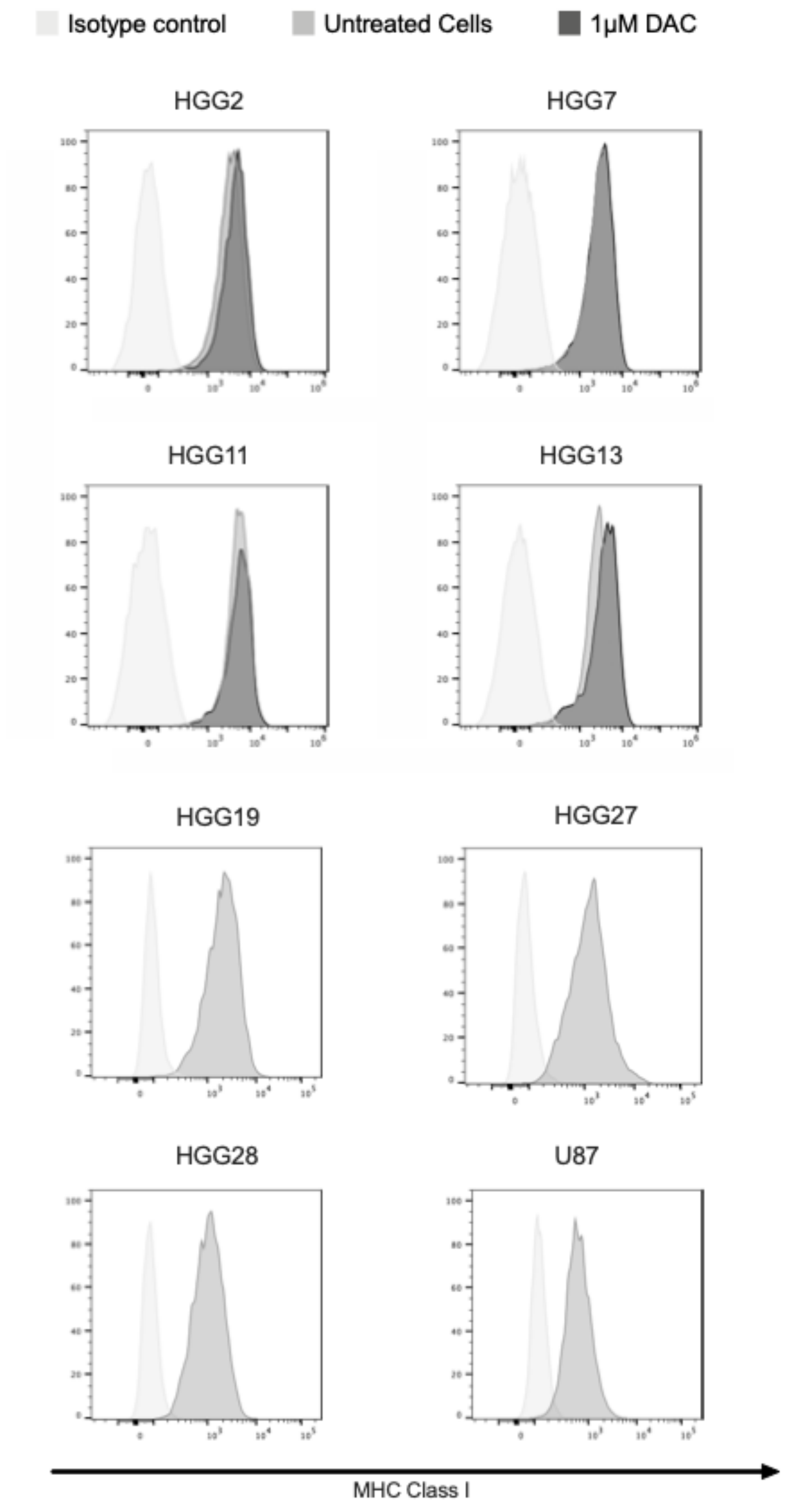

Supplementary figure 16. MHC Class I expression is high and unchanged following DAC treatment. Representative histograms, obtained by flow cytometry using a pan-class I antibody (clone W6/32) on HGG2,7,11,13,19,27,28,U87 cell lines show high level of baseline expression, which is unchanged following treatment with DAC (HGG2,7,11,13). Representative data from biological duplicates.

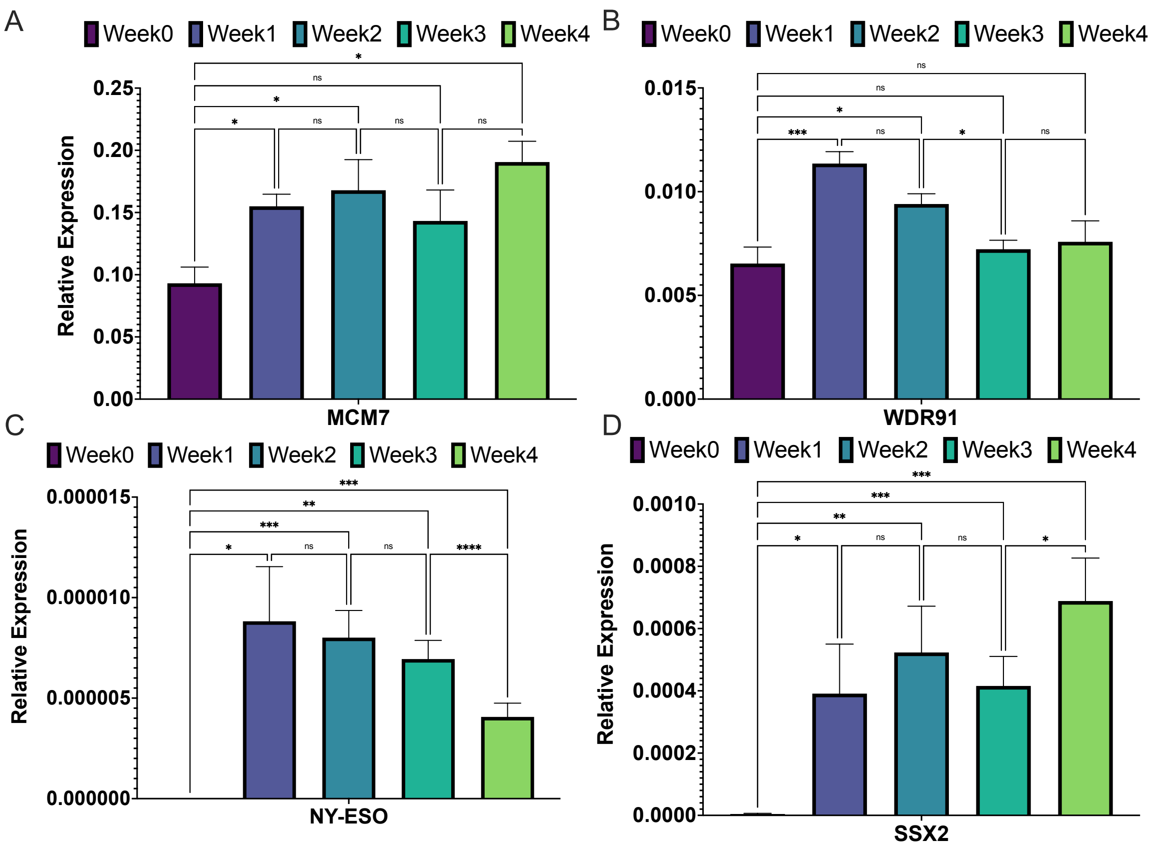

Supplementary figure 17. Increased gene expression following treatment with DAC varies but persists for a minimum of 2 weeks. Bar chart showing relative expression of genes with potential neoantigen encoding mutations or cancer testis antigens to GAPDH as measured by RTqPCR. Data generated for biological triplicates of HGG2 cell line. Expression is represented as $2^{-\Delta\Delta CT}$ and statistical significance as calculated by 2-way Anova. * p≤0.05, ** p≤0.01, ***p≤0.001, **** p≤0.0001

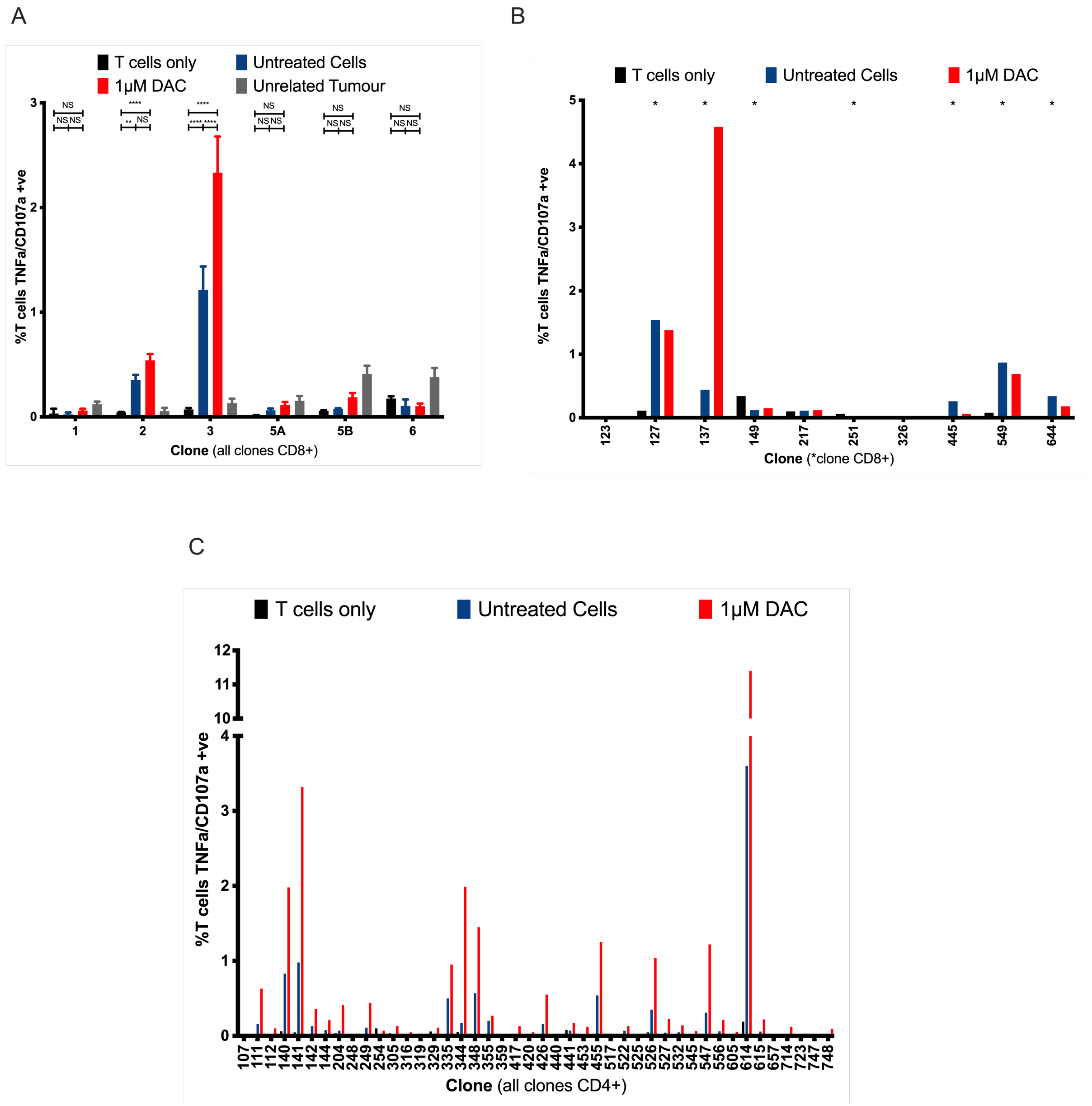

Supplementary figure 18. Tumour specific T cells can be isolated following MLTC and some show increased reactivity to autologous primary GBM cell line treated with DAC. Bar charts showing screen of T cell clones isolated following MLTC of (A) HGG19, (B) HGG2 and (c) HGG13. Activation of T cells following co-culture with autologous GBM cell line as measured by TNFα+/CD107a+ cells by flow cytometry. All cells pre-gated on CD3+/CD8+ or CD3+/CD4+ population depending on cell type. Data from (A) shown from 3 technical replicates, (B,C) single technical replicate, representative of a minimum of 2 biological replicates. Significance as determined by 2-way ANOVA. * p≤0.05, ** p≤0.01, ***p≤0.001, **** p≤0.0001

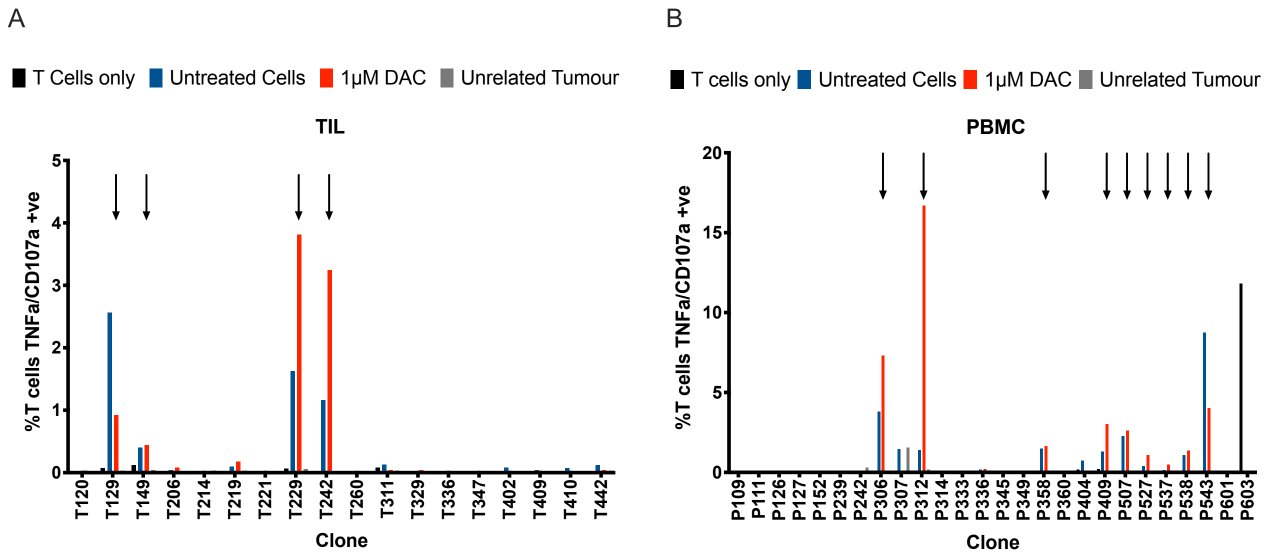

Supplementary figure 19. Tumour specific T cells can be isolated following single cell *ex-vivo* expansion and some show increased reactivity to autologous primary GBM cell line treated with DAC. Bar charts showing screen of T cell clones isolated following single cell expansion of (A) TIL and (B) PBMC. Activation of T cells following co-culture with autologous GBM cell line as measured by TNFα+/CD107a+ cells by flow cytometry. All cells pre-gated on CD3+/CD8+ or CD3+/CD4+ population depending on cell type. Data from single technical replicate representative of 2 biological replicates.
