# Supplementary material for: Decitabine increases neoantigen and cancer testis antigen expression to enhance T-cell–mediated toxicity against glioblastoma
Source: Neuro Oncol. 2022 Apr 25;24(12):2093–106. doi: 10.1093/neuonc/noac107 (PMC9713507; doi:10.1093/neuonc/noac107)
Supplement: noac107_suppl_Supplementary_Material [file noac107_suppl_supplementary_material.docx]

**Materials and methods**

*Patients and human samples*

All human samples were collected through the Oxford Brain Bank, approved by the local research ethics committee (15/SC/0639). Specific informed consent was mandatory for each patient and was obtained before each surgery for patients undergoing multiple operations. All patients were >18 years old and undergoing surgery for a radiologically diagnosed diffuse glioma. For each patient, tumour samples and blood were obtained at the time of surgery. All tumour samples were refrigerated during transportation and processed within 2h of surgery. Tumour samples were first washed in PBS, blood vessels and necrotic tissue were removed, and the samples were mechanically dissociated into small pieces (<2mm in size). This material was then digested enzymatically using the human tumour dissociation kit (Miltenyi) as per manufacturer’s instructions. The tumour digests underwent myelin removal and CD45 separation using MACS beads and columns (Miltenyi) as per manufacturer’s instructions. The CD45- fraction was used for establishment of primary tumour cell lines. Peripheral blood mononuclear cells (PBMC) were isolated from the fresh blood by centrifugation on Lymphoprep (Stem Cell Technologies) according to manufacturer’s instructions. HLA haplotyping was performed by the WIMM Sequencing Facility.

*Cell culture*

Primary tumour cell lines were cultured in serum-free media (RHB-A, Takara) supplemented with 20ng/mL epidermal growth factor, 20ng/mL fibroblast growth factor (Peprotech), and 100U/mL penicillin-0.1mg/mL streptomycin (Pen-Strep, Sigma). Tissue culture vessels were coated with laminin (Sigma) at 10μg/mL for a minimum of 2h before use. Cells were regularly observed and split 1:3-1:5 using accutase (BioLegend) when near confluent. All experiments with primary cell lines were done using early passage cells (passage<15). U87MG, HEK293T and normal human astrocytes (NHA) were cultured in DMEM (Sigma) supplemented with 10% foetal calf serum (FCS, Gibco) and Pen-Strep (D10). Primary immune cells were cultured in a base media of RPMI supplemented with 5% human serum (pooled serum from 10 different donors obtained from NHS blood and transplant services), 2mM L-Glutamine, 1x non-essential amino acids, 10mM HEPES, 1mM sodium pyruvate, Pen-Strep and 50µM 2-mecaptoethanol (RHS5). For T cell maintenance 500-1000IU IL-2 (produced in-house from J588L cell line[1]) was added to RHS5 (RHS5+IL-2). Additional cytokines were added to this media when appropriate as described.

Single T cell clonal expansion was performed by sorting single T cells into 96-well tissue culture plates loaded with 1.25x10^5^ irradiated allogeneic feeder cells per well and 1µg/mL of phytohaemagglutinin (PHA; Remel) in RHS5+IL2. Bulk T cell expansion was achieved by sorting T cells in bulk and expanding in RHS5+IL2 and PHA with irradiated feeder cells in a ratio of 200:1. Irradiated feeder cells were prepared by irradiating allogeneic PBMC from a minimum of 2 donors with 30Gy and pooling them in a ratio of 1:1.

## *In vitro decitabine treatment*

## Cells plated on the previous day were treated with fresh media containing the required concentration of DAC every 24h, for a total of 48h. Cells were then cultured with fresh media without decitabine for a further 3 days for transcriptomic analysis or 5 days for proteomic and functional assays. A paired sample was treated in an identical way without decitabine as an untreated control.

## *Isolation of neoantigen-specific T cells from patients*

Peptide stimulation was performed as previously described[2]. Briefly, patient PBMC were cultured in RHS5 supplemented with 10µg/mL of IL-7 (Peprotech) and stimulated with 30µM peptide pool. Three days later half of the media was replaced by RHS5+IL2. Cells were subsequently monitored and split as required over the next 2 weeks. Neoantigen-specific T cells were isolated using MHC-peptide tetramers as previously described[3]. Briefly, HLA-A2 monomers refolded with a photocleavable peptide (﻿KILGFVFJV, Pepscan) underwent peptide exchange under UV-light for 1h in excess concentration of neoantigen peptide. Monomers were multimerised using streptavidin-PE/streptavidin-APC (eBioscience) and free biotin was blocked with D-biotin. Cells were incubated with tetramers for 30min at 37°C and then stained with the antibodies of interest. CD3/CD8/tetramer+ cells were sorted and further expanded.

## *Generation of neoantigen-specific T cells from healthy donors*

Healthy donor T cells were primed *in-vitro* using a modified version of a previously described technique[4]. Briefly, monocytes from HLA-A2+ healthy donors were isolated using CD14 magnetic beads (Miltenyi) and differentiated in R10 supplemented with 1000IU IL-4 (produced in house from J588L cell line[1]), and 50ng/mL human GM-CSF (Peprotech) for 4 days. Cells were then matured with 2ng/mL TNFα (Peprotech) and 10ng/mL lipopolysaccharide (LPS Salmonella abortus equi, Sigma) for 24 hours. These monocyte-derived dendritic cells (moDCs) were split evenly and pulsed with 1µM of each individual peptide separately. Pulsed cells were then washed twice in PBS, pooled into patient-specific peptide pools, and mixed with the CD14- fraction at a ratio of 1:10 in RHS5 supplemented with IL-7 (10ng/mL) and IL-12 (50pg/mL) for 10-14 days with media changed on day 7. The cells were then re-stimulated with peptide-pulsed moDCs at a ratio of 1:5 in RHS5+IL2. These cells were then cultured for a further 2 weeks and split as required. CD3/CD8/tetramer+ cells were sorted and further expanded.

*Mixed tumour lymphocyte culture (MLTC)*

Patient PBMC were co-cultured with irradiated autologous primary tumour cell line (30Gy) for 10-14 days, in RHS5 supplemented with 10µg/mL IL-7 and 50pg/mL IL-12 (Peprotech) at a ratio of 1:5, with half of the media changed after 7 days. The cells were then re-stimulated with additional irradiated tumour cells for 14 days, at a ratio of 1:5 in RHS5+IL-2 and split as required. Tumour reactivity was measured by intracellular cytokine staining (ICS). Cells were stained with interferon-γ (IFN-γ) and tumour necrosis factor-α (TNF-α) capture assays (Miltenyi) according to manufacturer’s instructions. Single cells were sorted on either CD3/IFN-γ+, CD3/TNF-α+ or CD3/IFN-γ/TNF-α+ gates for both TCR sequencing and clonal expansion.

*Single cell ex-vivo expansion of T cell clones*

Fresh TIL and PBMC were sorted by FACS, gating on TCRβ+, into 96-well tissue culture plates loaded with 1 blood cell and 1.25x10^5^ irradiated allogeneic feeder cells per well in 150µL RHS5 supplemented with 1µg/mL PHA. A further 100µL RHS5 was added on day 3. Clones that had expanded sufficiently were passaged into 48- and then 24-well plates from day 14-21 as required. Clones that had grown sufficiently to be passaged and used for initial screening were then restimulated and expanded in RHS5+IL2 and PHA with irradiated feeder cells for further expansion.

*TCR sequencing and transduction*

Individual or 50 (mini-bulk) T cells were sorted into lysis buffer containing Triton X-100 (Sigma), RNAse inhibitor (Takara), dNTP (NEB), oligo(dT) primer, and TCRα/β-specific primers (IDT). Plates were snap frozen. Reverse transcription was performed with SMART Scribe reverse transcriptase (Takara) and a template switch oligo. Total cDNA was amplified by PCR using SeqAmp DNA polymerase (Takara). The PCR product was then used for targeted amplification of the TCRα/β chains in two subsequent rounds of nested PCR, using Phusion High Fidelity DNA polymerase (NEB). Indexes were added during the first PCR. One last PCR was performed to add Illumina adaptors. The final library was sequenced using the Miseq Reagent Kit V2 300 cycle in the MiSeq platform (Illumina). FASTQ files for each plate were obtained from BaseSpace (Illumina). Each FASTQ file was demultiplexed by exact barcode match. TCR sequences were analysed using MiXCR[5] and further downstream analysis was performed using VDJtools[6].

TCRs of interest were cloned into a pHR-SIN plasmid with the mouse TCRα/β constant regions. The TCRα and TCRβ VDJ regions were amplified by PCR from the DNA generated during TCR sequencing library preparation. Amplified TCR chains were purified using magnetic beads (AMPure XP, Beckman Coulter) and cloned in the plasmid using HiFi DNA assembly cloning kit

(NEB). Successfully cloned plasmids were confirmed by Sanger sequencing (Source Bioscience).

Lentivirus production was achieved by transfecting HEK 293T cells in 6-well plates with 4.5µL TurboFectin transfection reagent (Origene), 0.5µg pCMV-dR8.91, 0.25µg pMDG-VSVG and 1µg of pHR-SIN-TCR plasmid. Viral supernatants were collected 48h after transfection and centrifuged to remove any cells. Lentivirus supernatants were used to coat Retronectin-treated plates (Takara) as per manufacturer’s instructions. Primary CD8 T cells previously activated during 2 days with CD3/CD28 Dynabeads (Thermo Fisher) in RHS5/2 supplemented with 10ng/mL IL-5 (Peprotech) were incubated in the lentivirus coated plates. Three days later, mouse TCRβ expression was assessed by flow cytometry.

### T cell functional assays

Cancer cells were plated in RHS5 and, when applicable, pulsed with varying amounts of peptides for 1 hour at 37°C and subsequently washed twice. Cancer cells were then incubated with MHC-class I/II blocking antibodies (W6/32 at 20µg/mL, Tu39/L243/26.5 at 40µg/mL respectively), if required, for 1h. T cells, previously cultured without IL-2 overnight, were co-cultured with cancer cells at varying effector: target ratios. To evaluate cytokine production and degranulation, cells were co-cultured for 5h in the presence of anti-CD107a antibody (H4A3, PerCP-Cy5.5, BioLegend). Protein Transport Inhibitor Cocktail (eBioscience) was added for the last 4h. Cell Stimulation Cocktail (eBioscience) was used to stimulate the positive control cells. Cells were then stained with Zombie Aqua (eBioscience) and antibodies against CD3, CD8, IFN-g, TNFα (Biolegend) using the Intracellular Fixation and Permeabilization Buffer Set (eBioscience) following manufacturer’s instructions and analysed by flow cytometry. To evaluate T cell killing capacity, cells were co-cultured for 16h. T cell killing was assessed by LDH release using the Cyto-tox nonradioactive cytotoxicity assay (Promega) as per manufacturer’s instructions, using an iMark microplate reader (Bio-Rad). Specific T cell killing was calculated using the following formula:

$$Specific Lysis= \frac{Experimental readout-T cell only-tumour cell only}{tumour cell+lysis buffer-tumour cell only}$$

## *Flow cytometry*

Data was acquired using Fortessa X-20, X-50 (BD), or Attune NxT (Life technologies) flow cytometers. Cell sorting was performed on either Fusion 2, Aria III (BD) or SH800 (Sony) cell sorters. Data was analysed using FlowJo. All antibodies and fluorescent dyes used are described in the table below. All targets are human, except when stated.

*Whole exome sequencing (WES)*

Genomic DNA was extracted from primary tumour samples, cell lines and PBMC (germ line) using DNEasy blood and tissue kit (Qiagen) as per manufacturer’s instructions. WES was performed by Novogene Co. Ltd using SureSelect Human All Exon V6 (Agilent) and Next Ultra DNA Library Prep Kit (NEB). The library was sequenced using the HiSeq platform (Illumina), utilising a paired-end 150-base-pair sequencing strategy. Sequencing was performed at a depth of 50X for PBMC and primary cell lines and 150X for tumour samples.

WES reads were processed, mapped and had somatic variants called using two concurrent workflows. In one workflow, reads were trimmed using Trim Galore v0.4.1 (Babraham Bioinformatics; utilising Cutadapt version [7] 1.2.1 and FastQC v0.10.1 (Babraham Bioinformatics)) and mapped against *Homo sapiens* genome version hg38 using Bowtie2 [7] v2.2.5. Duplicate reads in mapped files were detected and removed using Picard MarkDuplicates (Broad Institute) v2.3.0 and the resulting mapped sequences were sorted and indexed using Samtools [8,9] v1.6. The other workflow used was GATK4 best practices. Finally, somatic variants were called using Strelka2 [10] and GATK’s Mutect2 (Broad institute) at the end of each respective workflow. For a final mutation list, the union of somatic variants called from either workflow with a variant allele frequency >5% in the tumour samples and <5% in the PBMC were considered for neoantigen prediction.

*RNA sequencing*

Cell lines were treated with decitabine as previously described, in three independent experiments. Untreated controls were prepared in parallel. Cells were harvested on day 5 and RNA was extracted using RNEasy kit (Qiagen) as per manufacturer’s instructions. 1µg RNA was sent for library preparation and sequencing by Novogene Co. Ltd. Library preparation was performed using Next® Ultra RNA Library Prep Kit for Illumina® (NEB) and sequenced using the NovaSeq platform (Illumina) with a paired-end 150-base-pair sequencing strategy.

RNA-Seq reads were processed using Trim Galore, Picard MarkDuplicates and Samtools and were mapped against Homo sapiens genome version hg38 using STAR [11] v2.5.3a. The featureCounts tool from the Subread package v1.6.2 was used to quantify reads to generate counts per gene, which were then transformed into transcripts per million (TPM) values using an in-house Python script. Differential gene expression analysis was performed using DEseq2 [12]. Gene set enrichment analysis (GSEA) was performed using clusterProfiler [13] using mSigDB curated gene sets [14–16]. Results were filtered with adjusted p<0.05. The top 10 gene sets were selected for display using Normalised Enrichment Value (NES) metric.

*Methylation sequencing*

Global methylation analysis was performed by Diagenode using Infinium methylation EPIC BeadChip Kit (Illumina). Analysis was performed using the ChAMP Bioconductor package [17,18]. Probes were removed if their detection P-value exceeded 0.01, and the β-values were normalised by peak-based correction[19]. The change in methylation between conditions was evaluated in a pairwise fashion. Differentially methylated probes were defined as a probe with an adjusted p-value <0.05 and a differentially methylated gene was defined as a gene with >1 differentially methylated probes. Candidate gene methylation data was then plotted using CandiMeth[20].

For targeted bisulphite sequencing, 2 µg of genomic DNA was bisulphite converted using the Zymo EZ DNA Methylation-Lightning Kit (Cambridge Bioscience, D5030) and loci in the promoter of several genes were specifically amplified using the PyroMark PCR kit (Qiagen, 978703) according to the manufacturer’s recommendations. The PCR primers used are listed in the table below and were modified to contain the non-variable part of Illumina multiplexing adaptors for subsequent sample barcoding. Following PCRs, the products were purified using 1.8x AMPure XP beads (Beckman Coulter, A63881) and quantified by fluorescence using the high sensitivity Quant-iT™ dsDNA Assay Kit (Thermo Fisher Scientific, Q33120) for equimolar pooling of all genes per sample. The samples were then individually barcoded by PCR using PyroMark PCR kit (Qiagen, 978703) and Illumina dual index primers, purified twice with 1.6x AMPure XP beads, quantified by Qubit dsDNA HS kit (Invitrogen, Q32851) before equimolar pooling of each sample and sequencing of the final library on MiSeq. The resultant reads were separated by gene according to primer sequence then corrected for sequencing errors, based on the expected sequence from the GRCh37 reference genome. A maximum of three indels of up to three nucleotides each were corrected if they immediately followed a repetitive region (≥5 nucleotides in which ≥80% of positions were the same base) and did not contain a CpG site. CpG sites, point mutations and more erroneous reads were not changed to avoid false corrections. Bismark was used to extract methylation data [21] and the data are displayed in graphs based on Methylation plotter [22].

*Neoantigen prediction and peptides*

Neoantigen prediction was performed by an in-house re-developed version of MuPeXI [23] codenamed TUNAPASTA v0.5, which follows much of the same rationale. Patient mutations, HLA-types and TPM expression data are input and processed to determine a ranked list of neoantigens. TUNAPASTA relies on the Ensembl Variant Effect Predictor (VEP) [24] v92.1, NetMHCpan [24] v4.0 and Python v3. Predicted neoantigen peptides were brought from Pepscan (Netherlands) and Genecust (France) at crude purity, resuspended in DMSO to 10mg/mL stock and stored at -80°C.

*Quantitative RT-qPCR*

RNA was extracted using RNeasy kit (Qiagen) and reverse transcription was performed using RETROscript (Invitrogen) or LunaScript (NEB). TaqMan probes (Life Technologies; see table below) and the TaqMan Fast Advanced Master Mix (Life Technologies) were used for the assay. qPCR was performed using the QuantStudio 7 (Life Technologies). ∆∆CT was calculated for a CT value of 0.02.

**Supplementary references**

1 Tunnacliffe A, Olsson C, Traunecker A, *et al.* Production and secretion of recombinant soluble CD3 polypeptides by myeloma-derived transfectant clones. *Immunol Lett* 1989;**21**:243–7. doi:10.1016/0165-2478(89)90111-9

2 Chen JL, Dawoodji A, Tarlton A, *et al.* NY-ESO-1 specific antibody and cellular responses in melanoma patients primed with NY-ESO-1 protein in ISCOMATRIX and boosted with recombinant NY-ESO-1 fowlpox virus. *Int J Cancer* 2015;**136**:E590–601. doi:10.1002/ijc.29118

3 Rodenko B, Toebes M, Hadrup SR, *et al.* Generation of peptide-MHC class I complexes through UV-mediated ligand exchange. *Nat Protoc* 2006;**1**:1120–32. doi:10.1038/nprot.2006.121

4 Ali M, Foldvari Z, Giannakopoulou E, *et al.* Induction of neoantigen-reactive T cells from healthy donors. *Nat Protoc* 2019;**14**. doi:10.1038/s41596-019-0170-6

5 Bolotin DA, Poslavsky S, Mitrophanov I, *et al.* MiXCR: software for comprehensive adaptive immunity profiling. *Nat Methods* 2015;**12**:380–1. doi:10.1038/nmeth.3364

6 Shugay M, Bagaev D V., Turchaninova MA, *et al.* VDJtools: Unifying Post-analysis of T Cell Receptor Repertoires. *PLOS Comput Biol* 2015;**11**:e1004503. doi:10.1371/journal.pcbi.1004503

7 Martin M. Cutadapt removes adapter sequences from high-throughput sequencing reads. *EMBnet.journal* 2011;**17**:10. doi:10.14806/ej.17.1.200

8 Li H, Handsaker B, Wysoker A, *et al.* The Sequence Alignment/Map format and SAMtools. *Bioinformatics* 2009;**25**:2078–9. doi:10.1093/bioinformatics/btp352

9 Li H. A statistical framework for SNP calling, mutation discovery, association mapping and population genetical parameter estimation from sequencing data. *Bioinformatics* 2011;**27**:2987–93. doi:10.1093/bioinformatics/btr509

10 Kim S, Scheffler K, Halpern AL, *et al.* Strelka2: fast and accurate calling of germline and somatic variants. *Nat Methods* 2018;**15**:591–4. doi:10.1038/s41592-018-0051-x

11 Dobin A, Davis CA, Schlesinger F, *et al.* STAR: ultrafast universal RNA-seq aligner. *Bioinformatics* 2013;**29**:15–21. doi:10.1093/bioinformatics/bts635

12 Love MI, Huber W, Anders S. Moderated estimation of fold change and dispersion for RNA-seq data with DESeq2. *Genome Biol* 2014;**15**:550. doi:10.1186/s13059-014-0550-8

13 Yu G, Wang L-G, Han Y, *et al.* clusterProfiler: an R Package for Comparing Biological Themes Among Gene Clusters. *Omi A J Integr Biol* 2012;**16**:284–7. doi:10.1089/omi.2011.0118

14 Subramanian A, Tamayo P, Mootha VK, *et al.* Gene set enrichment analysis: a knowledge-based approach for interpreting genome-wide expression profiles. *Proc Natl Acad Sci U S A* 2005;**102**:15545–50. doi:10.1073/pnas.0506580102

15 Liberzon A, Subramanian A, Pinchback R, *et al.* Molecular signatures database (MSigDB) 3.0. *Bioinformatics* 2011;**27**:1739–40. doi:10.1093/bioinformatics/btr260

16 Liberzon A, Birger C, Thorvaldsdóttir H, *et al.* The Molecular Signatures Database (MSigDB) hallmark gene set collection. *Cell Syst* 2015;**1**:417–25. doi:10.1016/j.cels.2015.12.004

17 Morris TJ, Butcher LM, Feber A, *et al.* ChAMP: 450k Chip Analysis Methylation Pipeline. *Bioinformatics* 2014;**30**:428–30. doi:10.1093/bioinformatics/btt684

18 Aryee MJ, Jaffe AE, Corrada-Bravo H, *et al.* Minfi: A flexible and comprehensive Bioconductor package for the analysis of Infinium DNA methylation microarrays. *Bioinformatics* 2014;**30**:1363–9. doi:10.1093/bioinformatics/btu049

19 Dedeurwaerder S, Defrance M, Calonne E, *et al.* Evaluation of the Infinium Methylation 450K technology. *Epigenomics* 2011;**3**:771–84. doi:10.2217/epi.11.105

20 Thursby S-J, Lobo DK, Pentieva K, *et al.* CandiMeth: Powerful yet simple visualization and quantification of DNA methylation at candidate genes. *Gigascience* 2020;**9**. doi:10.1093/gigascience/giaa066

21 Krueger F, Andrews SR. Bismark: A flexible aligner and methylation caller for Bisulfite-Seq applications. *Bioinformatics* 2011;**27**:1571–2. doi:10.1093/bioinformatics/btr167

22 Mallona I, Díez-Villanueva A, Peinado MA. Methylation plotter: A web tool for dynamic visualization of DNA methylation data. Source Code Biol. Med. 2014;**9**:11. doi:10.1186/1751-0473-9-11

23 Bjerregaard A-M, Nielsen M, Hadrup SR, *et al.* MuPeXI: prediction of neo-epitopes from tumor sequencing data. *Cancer Immunol Immunother* 2017;**66**:1123–30. doi:10.1007/s00262-017-2001-3

24 Jurtz V, Paul S, Andreatta M, *et al.* NetMHCpan-4.0: Improved Peptide–MHC Class I Interaction Predictions Integrating Eluted Ligand and Peptide Binding Affinity Data. *J Immunol* 2017;**199**:3360–8. doi:10.4049/jimmunol.1700893

25 Morris TJ, Butcher LM, Feber A, *et al.* ChAMP: 450k Chip Analysis Methylation Pipeline. *Bioinformatics* 2014;**30**:428–30. doi:10.1093/bioinformatics/btt684
