# Supplementary material for: Decitabine increases neoantigen and cancer testis antigen expression to enhance T-cell–mediated toxicity against glioblastoma
Source: Neuro Oncol. 2022 Apr 25;24(12):2093–106. doi: 10.1093/neuonc/noac107 (PMC9713507; doi:10.1093/neuonc/noac107)
Supplement: noac107_suppl_Supplementary_Tables [file noac107_suppl_supplementary_tables.docx]

| **Target** | **Clone** | **Fluorochrome** | **Company** | **Product code** |
| --- | --- | --- | --- | --- |
| CD107a | H4A3 | PerCP-Cy5.5 | BioLegend | 328616 |
| CD3 | HIT1a | PE-Cy7 | BioLegend | 300316 |
| CD4 | RPA-T4 | FITC | BD | 555346 |
| CD4 | OKT4 | APC | BioLegend | 317416 |
| CD4 | OKT4 | APC-Cy7 | BioLegend | 317418 |
| CD8 | SK1 | BV711 | BioLegend | 344734 |
| CD8 | SK1 | PerCP-Cy5.5 | BioLegend | 344710 |
| CD8 | SK1 | APC-Cy7 | BioLegend | 344713 |
| CD8a | HIT8a | PE-Cy7 | BioLegend | 300914 |
| HLA-A2 | BB7.2 | PE | BioLegend | 343305 |
| HLA-DR | TU36 | PE | BioLegend | 361605 |
| TCRβ | T10B9 | FITC | BD | 555547 |
| IFN-γ | 4s.B3 | APC-Cy7 | Invitrogen | 47-7319-42 |
| IFN-γ | 4s.B3 | APC | BioLegend | 502512 |
| MHC class I | W6/32 | PE | BioLegend | 311405 |
| MHC class I | W6/32 | Purified | BioLegend | 311428 |
| Mouse TCRβ | H57-597 | APC-Cy7 | BioLegend | 109220 |
| NESTIN | 196908 | PE | R&D Systems | IC1259P |
| NG2 | LHM-2 | APC | BioLegend | FAB2585A |
| NY-ESO | E978 | Purified | Sigma | N2038 |
| PD1 | eBioJ105 | PE-Cy7 | eBioscience | 25-2799-41 |
| SLC6A12 | Polyclonal | Purified | Life Technologies | PA524005 |
| Streptavidin |  | PE | BioLegend | 12-4317-87 |
| Streptavidin |  | APC | BioLegend | 17-4317-82 |
| TNF-α | MAb11 | PE | BioLegend | 502909 |
| Zombie Aqua |  |  | BioLegend | 77143 |

**Supplementary table 1. List of antibodies and fluorescent dyes used.**

| **Gene name** | **Illumina adaptor and PCR primer sequence (5'-3')** |
| --- | --- |
| MCM7 Fwd | **ACACTCTTTCCCTACACGACGCTCTTCCGATCT**GGTTTTTTAAAGTGTTGGGATTATAG |
| MCM7 Rev | **GTGACTGGAGTTCAGACGTGTGCTCTTCCGATCT**CTCCCTCCCAAAACTAATTCATTA |
| WDR91 Fwd | **ACACTCTTTCCCTACACGACGCTCTTCCGATCT**TTGTTTTTAGGGTTTTAGTTTTTTT |
| WDR91 Rev | **GTGACTGGAGTTCAGACGTGTGCTCTTCCGATCT**ATCTTAAAAACATCCCAAATAATCC |
| DNAH3 Fwd | **ACACTCTTTCCCTACACGACGCTCTTCCGATCT**AGGAGGGGTTATATTTTTGGTTG |
| DNAH3 Rev | **GTGACTGGAGTTCAGACGTGTGCTCTTCCGATCT**CTCCTCTCCTTAAATCTCCCTAACT |
| PRAME Fwd | **ACACTCTTTCCCTACACGACGCTCTTCCGATCT**TTTTGAATGTAGGGAAAGTAGGG |
| PRAME Rev | **GTGACTGGAGTTCAGACGTGTGCTCTTCCGATCT**ATTTCCCAAAACTTTCTAAAACCC |
| MAGEA1 Fwd | **ACACTCTTTCCCTACACGACGCTCTTCCGATCT**TTATTTTTATTTTTATTTTTATTTTTA |
| MAGEA1 Rev | **GTGACTGGAGTTCAGACGTGTGCTCTTCCGATCT**AAACCTAAATCAAATTCCTTC |
| SSX2 Fwd | **ACACTCTTTCCCTACACGACGCTCTTCCGATCT**GGGTGGTGAATGTTTGTAGTTT |
| SSX2 Rev | **GTGACTGGAGTTCAGACGTGTGCTCTTCCGATCT**CTACCTTAACCAATCCTCCAAC |
| FAM122a Fwd | **ACACTCTTTCCCTACACGACGCTCTTCCGATCT**AGAAGATGGAGTTAGATTTGGAGTT |
| FAM122a Rev | **GTGACTGGAGTTCAGACGTGTGCTCTTCCGATCT**CTATTAAACCTCCTAAAACCCCC |
| CTAG1B Fwd | **ACACTCTTTCCCTACACGACGCTCTTCCGATCT**GTATTTTTGATGGTTTAGGGGGTA |
| CTAG1B Rev | **GTGACTGGAGTTCAGACGTGTGCTCTTCCGATCT**ATCTACAACATCCATTCAACCCTA |
| MAGEA1 Fwd | **ACTCTTTCCCTACACGACGCTCTTCCGATCT**TTTTATTTTTATTTTTATTTTTATTTTTA |
| MAGEA1 Rev | **GTGACTGGAGTTCAGACGTGTGCTCTTCCGATCT**CACAAAACCTAAATCAAATTCCTTC |

**Supplementary table 2. List of PCR primers for PCR amplification of bisulphite-treated loci**

| **Gene** | **Reference** |
| --- | --- |
| LAMA3 | Hs00165042_m1 |
| FAM122a | Hs00540770_s1 |
| WDR91 | Hs00608466_m1 |
| DNAH3 | Hs00954323_m1 |
| SSX2 | Hs00817683_m1 |
| NY-ESO | Hs00265824_m1 |
| GAPDH | Hs02758991_g1 |
| MYRF | Hs00973739_m1 |
| DNAH9 | Hs01017795_m1 |
| EPHA3 | Hs00739092_m1 |
| CARD14 | Hs01106900_m1 |
| PTPN5 | Hs00377920_m1 |
| SLC6A12 | Hs00758246_m1 |
| OTOF | Hs00191271_m1 |
| OR2D2 | Hs00999189_s1 |
| MASP1 | Hs00373559_m1 |
| MCM7 | Hs00428518_m1 |

**Supplementary table 3. List of TaqMan Probes used.**

| **Gene** | **Chromosome** | **Position** | **Reference** | **Mutation** |
| --- | --- | --- | --- | --- |
| MYRF | 11 | 61541446 | C | T |
| DNAH9 | 17 | 11837216 | G | A |
| EPHA3 | 3 | 89448536 | G | T |
| OTOF | 2 | 26684695 | G | A |
| SLC6A12 | 12 | 313816 | G | A |
| MASP1 | 3 | 186944297 | T | G |
| OR2D2 | 11 | 6913503 | T | C |
| CARD14 | 17 | 78164655 | C | T |
| PTPN5 | 11 | 18754846 | C | T |

**Supplementary table 4. Top 9 potential neoantigen mutations identified from U87MG**. Mutation data was obtained from CCLE and neoantigens predicted from this. The top neoantigens as determined by HLA-A2 restricted priority score were selected and the mutations giving rise to these neoantigens are shown.

**(A)**

| **Patient ID** | **Age** | **Primary/ Secondary GBM** | **IDH** | **MGMT status** | **OS  (months)** |  |
| --- | --- | --- | --- | --- | --- | --- |
|  |  |  |  |  |  |  |
| **HGG2** | 58 | Recurrent | WT | - | 7.4 |  |
| **HGG7** | 66 | Primary | WT | - | 10.4 |  |
| **HGG11** | 66 | Primary | WT | - | 12.7 |  |
| **HGG13** | 59 | Primary | WT | - | 24.7 |  |
| **HGG19** | 68 | Primary | WT | - | 10.4 |  |
| **HGG27** | 79 | Primary | WT | hypermethylated | 6.3 |  |
| **HGG28** | 58 | Primary | WT | hypermethylated | 9.5 |  |
| **HGG37** | 59 | Primary | WT | unmethylated | 29.5 |  |

**(B)**

| **Cell line** | **MHC Class I alleles** | | | | | |
| --- | --- | --- | --- | --- | --- | --- |
|  | **A1** | **A2** | **B1** | **B2** | **Cw1** | **Cw2** |
| **HGG2** | 02:01 | 24:02 | 18:01 | 44:02 | 05:01/03 | 12:03 |
| **HGG7** | 02:01 | 02:01 | 40:01 | 40:01 | 03:04 | 03:04 |
| **HGG11** | 02:01 | 68:02 | 44:02 | 53:01 | 04:01 | 05:01/03 |
| **HGG13** | 01:01/04N | 02:01 | 07:02 | 08:01 | 07:01 | 07:02 |
| **HGG19** | 02:01 | 03:01 | 07:02 | 52:01 | 07:02 | 12:02 |
| **HGG27** | 03:01 | 68:01 | 07:02 | 15:01 | 04:01 | 07:02 |
| **HGG28** | 03:01 | 32:01 | 07:02 | 44:03 | 07:02 | 16:01 |
| **HGG37** | 01:01 | 01:01 | 40:02 | 40:02 | 07:01 | 07:01 |
| **U87** | 02:01 | 02:01 | 44:02 | 44:02 | 05:01/03 | 05:01/03 |

**Supplementary table 5.** (A) Key Clinical information and (B) MHC class I haplotypes of patients from whom primary cell lines were derived and used for the study.

| **Patient ID** | **Peptide** | **Gene** | **Mutation** | **Designation** |
| --- | --- | --- | --- | --- |
| HGG2 | FLEEIILKSL | BDP1 | chr5.71553194.G>T | H2A1 |
| HGG2 | FLRESQNPL | GRIN3A | chr9.101737294.G>A | H2A2 |
| HGG2 | GLALGTPLSI | AMER3 | chr2.130763424.C>T | H2A3 |
| HGG2 | GLAVNLSQI | KIAA1211 | chr4.56315319.C>T | H2A4 |
| HGG2 | GNLPDIEVRL | RP5 | chr17.40656487.T>C | H2A5 |
| HGG2 | LLQDGRTLI | LSM1 | chr8.38172012.C>T | H2A6 |
| HGG2 | KEMTDITIKA | ADGRV1 | chr5.90674196.C>T | H2A7 |
| HGG2 | TLCVADFGL | TYRO3 | chr15.41573327.G>T | H2A8 |
| HGG2 | RLWPSSWVS | ADRA1D | NA | H2A9 |
| HGG2 | VLAADIQQC | FSIP2 | chr2.185738977.C>T | H2A10 |
| HGG2 | FLRESQNPLHL | GRIN3A | chr9.101737294.G>A | H2A11 |
| HGG2 | AVEDELLQAV | KBTBD13 | chr15.65077036.C>T | H2A12 |
| HGG2 | TMLISILKL | C6orf120 | chr6.169702993.A>G | H2B1 |
| HGG2 | YILVDIDDTF | RP11 | chr10.73807430.A>G | H2B2 |
| HGG2 | KTLSHLQTL | CD180 | chr5.67183662.T>G | H2B3 |
| HGG2 | RLWPSSWV | ADRA1D | NA | H2B4 |
| HGG2 | NLAEAQSAA | GRB10 | chr7.50605336.G>A | H2B5 |
| HGG2 | FVMRNHDVV | C9 | chr5.39311316.C>T | H2B6 |
| HGG2 | HQFNLAETL | MIER2 | chr19.334497.A>G | H2B7 |
| HGG2 | QVFDFKLSDV | AKR1B15 | chr7.134577729.A>T | H2B8 |
| HGG2 | LLALMTVAM | PTGIR | chr19.46623486.A>G | H2B9 |
| HGG2 | NLAEAQSAAL | GRB10 | chr7.50605336.G>A | H2B10 |
| HGG2 | LVSDVSVDSV | VWF | chr12.6016143.T>A | H2B11 |
| HGG2 | MLAEDMTLCVA | TYRO3 | chr15.41573327.G>T | H2B12 |
| HGG2 | KLDYLEEKA | CMYA5 | chr5.79737699.A>T | H2C1 |
| HGG2 | VLLQDGRTL | LSM1 | chr8.38172012.C>T | H2C2 |
| HGG2 | AALAGGLYEYV | AQP4 | chr18.26856458.A>G | H2C3 |
| HGG2 | YITAAYVEV | MCM7 | chr7.100094259.T>C | H2C4 |
| HGG2 | LLLERGYDVNL | TANC1 | chr2.159219266.G>A | H2C5 |
| HGG2 | EMTDITIKA | ADGRV1 | chr5.90674196.C>T | H2C6 |
| HGG2 | ALAGGLYEYVF | AQP4 | chr18.26856458.A>G | H2C7 |
| HGG2 | ALAGGLYEY | AQP4 | chr18.26856458.A>G | H2C8 |
| HGG2 | ALFCHQYDI | FNDC5 | chr1.32864776.C>T | H2C9 |
| HGG2 | ILVSDVSVDSV | VWF | chr12.6016143.T>A | H2C10 |
| HGG2 | AVAQDYAQGV | SIPA1L3 | chr19.38119638.T>C | H2C11 |
| HGG2 | RLLLERGYDV | TANC1 | chr2.159219266.G>A | H2C12 |
| HGG2 | FKLSDVEMAT | AKR1B15 | chr7.134577729.A>T | H2D1 |
| HGG2 | ILVSDVSV | VWF | chr12.6016143.T>A | H2D2 |
| HGG2 | ALQQLTTHM | TSHZ2 | chr20.53254629.A>G | H2D3 |
| HGG2 | ILFQCMPVPV | WDR91 | chr7.135208809.C>T | H2D4 |
| HGG2 | VVVYGMEYL | ECE2 | chr3.184285061.T>A | H2D5 |
| HGG2 | GRVIENLAEA | GRB10 | chr7.50605336.G>A | H2D6 |
| HGG2 | LLLERGYDV | TANC1 | chr2.159219266.G>A | H2D7 |
| HGG2 | SVATGDVIIFL | GALNT10 | chr5.154376409.C>T | H2D8 |
| HGG2 | ILFSAAKHSV | COMMD4 | chr15.75339033.C>T | H2D9 |
| HGG2 | FVMRNHDVVL | C9 | chr5.39311316.C>T | H2D10 |
| HGG2 | ILNKTSGPMEV | POLR1E | chr9.37489350.A>C | H2D11 |
| HGG2 | IIPPMFGTV | ACER3 | chr11.76926604.G>A | H2D12 |
| HGG2 | ILVDIDDTF | RP11 | chr10.73807430.A>G | H2E1 |
| HGG2 | YLLTGQQKT | DLX6 | chr7.97007638.A>G | H2E2 |
| HGG2 | KLSDVEMAT | AKR1B15 | chr7.134577729.A>T | H2E3 |
| HGG7 | AAIESFVSV | ALOXE3 | chr17.8108578.T>A | H7A1 |
| HGG7 | LMAPLSPGA | SRRT | NA | H7A2 |
| HGG7 | LEMDDFEIVV | KCNJ18 | chr17.21703655.C>T | H7A3 |
| HGG7 | GMLKTDEGI | PRDX2 | chr19.12800208.C>T | H7A4 |
| HGG7 | EMDDFEIVV | KCNJ18 | chr17.21703655.C>T | H7A5 |
| HGG7 | LLMRYLKAI | LTN1 | NA | H7A6 |
| HGG7 | LLLHSSNLI | CCDC150 | NA | H7A7 |
| HGG7 | AMGNELIQVL | RFX8 | chr2.101402438.T>C | H7A8 |
| HGG7 | GLSSVALAFL | ABCA8 | chr17.68929562.A>G | H7A9 |
| HGG7 | KMVRLDLLM | LTN1 | NA | H7A10 |
| HGG7 | ALTGLVFL | SLC6A18 | chr5.1244223.G>T | H7A11 |
| HGG7 | MMVNFVVEC | OR5R1 | chr11.56417968.G>A | H7A12 |
| HGG7 | TAMGNELIQV | RFX8 | chr2.101402438.T>C | H7B1 |
| HGG7 | QLMAPLSPGA | SRRT | NA | H7B2 |
| HGG7 | ALSTVRVGA | CLEC14A | chr14.38255826.G>A | H7B3 |
| HGG7 | ILWFEAGEISL | OR51F1 | chr11.4769644.G>C | H7B4 |
| HGG7 | SQIFFSLHL | OR7C1 | NA | H7B5 |
| HGG7 | TLTSWSLAA | ATAD3B | chr1.1495668.A>G | H7B6 |
| HGG7 | SLFLLYGLSSV | ABCA8 | chr17.68929562.A>G | H7B7 |
| HGG7 | RLDLLMRYL | LTN1 | NA | H7B8 |
| HGG7 | SLHLDAWTI | OR7C1 | NA | H7B9 |
| HGG7 | RLLLHSSNL | CCDC150 | NA | H7B10 |
| HGG7 | TIWVDLLRA | PARG | chr10.49932102.G>A | H7B11 |
| HGG7 | HLDAWTIYS | OR7C1 | NA | H7B12 |
| HGG7 | FLLYGLSSVAL | ABCA8 | chr17.68929562.A>G | H7C1 |
| HGG7 | LKLLAEELNQL | PSTK | chr10.122990224.A>C | H7C2 |
| HGG7 | GLSSVALAF | ABCA8 | chr17.68929562.A>G | H7C3 |
| HGG7 | KIEGGIHTA | RTCA | chr1.100268258.A>G | H7C4 |
| HGG7 | LFLLYGLSSV | ABCA8 | chr17.68929562.A>G | H7C5 |
| HGG7 | LLYGLSSV | ABCA8 | chr17.68929562.A>G | H7C6 |
| HGG7 | LLAEELNQLKA | PSTK | chr10.122990224.A>C | H7C7 |
| HGG7 | VLLAPPATGM | PLXNA2 | chr1.208217826.C>T | H7C8 |
| HGG7 | TIMNTQLWV | OR4D2 | chr17.58170058.G>A | H7C9 |
| HGG7 | RLSEDYGML | PRDX2 | chr19.12800208.C>T | H7C10 |
| HGG11 | WLSEDEIML | PTEN | NA | H11A1 |
| HGG11 | YLSAIMPSQL | FIGN | chr2.163609698.C>A | H11A2 |
| HGG11 | LLAPFSEEGL | E4F1 | chr16.2223750.C>T | H11A3 |
| HGG11 | VLLAMAFDHYV | OR51E1 | chr11.4652900.G>A | H11A4 |
| HGG11 | LAMAFDHYV | OR51E1 | chr11.4652900.G>A | H11A5 |
| HGG11 | QWLSEDEIML | PTEN | NA | H11A6 |
| HGG11 | ALAPSGFLGLL | E4F1 | chr16.2223750.C>T | H11A7 |
| HGG11 | YLDEPFWAQ | SCN10A | chr3.38750124.G>A | H11A8 |
| HGG11 | ALKSDFKLV | MAML3 | NA | H11A9 |
| HGG11 | ILPDFLPI | PAK5 | NA | H11A10 |
| HGG11 | VLLYCIAAL | SYPL1 | chr7.106097768.G>T | H11A11 |
| HGG11 | FLEAFHLV | PPEF2 | chr4.75860764.C>T | H11A12 |
| HGG11 | YIRFFITYV | FADS2 | chr11.61863020.A>G | H11B1 |
| HGG11 | IIFFFLNRV | ZNF880 | NA | H11B2 |
| HGG11 | VLSSVNSNLL | NOX1 | chrX.100843369.G>T | H11B3 |
| HGG11 | KVDHKGPQI | AHNAK2 | chr14.104945729.C>T | H11B4 |
| HGG11 | DIVGFFFSV | GK5 | NA | H11B5 |
| HGG11 | MLDSRSTNRV | KLHDC2 | chr14.49777866.G>A | H11B6 |
| HGG11 | ILPDFLPII | PAK5 | NA | H11B7 |
| HGG11 | MLQQFTVKL | PTEN | NA | H11B8 |
| HGG11 | FLPIITHI | PAK5 | NA | H11B9 |
| HGG11 | KLVSLRTSL | MAML3 | NA | H11B10 |
| HGG11 | LVELDETSQEL | DLGAP5 | chr14.55188974.C>T | H11B11 |
| HGG11 | LLAMAFDHYV | OR51E1 | chr11.4652900.G>A | H11B12 |
| HGG11 | IMLQQFTVKL | PTEN | NA | H11C1 |
| HGG11 | SLFFFFGV | CHL1 | NA | H11C2 |
| HGG11 | GLLAPFSEEGL | E4F1 | chr16.2223750.C>T | H11C3 |
| HGG11 | KLPDLWCKI | LAMA3 | chr18.23909188.C>T | H11C4 |
| HGG11 | FLNILYPKT | SLITRK4 | chrX.143630878.A>C | H11C5 |
| HGG11 | ALQEEQNIL | CCDC7 | chr10.32456320.A>C | H11C6 |
| HGG11 | FLLICGLQQV | GLT1D1 | chr12.128926413.G>A | H11C7 |
| HGG11 | ELDETSQEL | DLGAP5 | chr14.55188974.C>T | H11C8 |
| HGG11 | REMIPAVLPL | AP4E1 | chr15.50925164.T>C | H11C9 |
| HGG11 | LLLGDMDQGI | WBSCR22 | chr7.73687050.G>A | H11C10 |
| HGG13 | YFCLSPPPTLL | DNAH2 | NA | H13A1 |
| HGG13 | IMEEVWYFL | DNAH3 | chr16.20963718.G>A | H13A2 |
| HGG13 | IVVEFSSPV | RARS2 | NA | H13A3 |
| HGG13 | LLPAHYETL | CHN2 | chr7.29509358.C>T | H13A4 |
| HGG13 | CLSPPPTLL | DNAH2 | NA | H13A5 |
| HGG13 | KVEEALVLL | EME2 | chr16.1775397.G>A | H13A6 |
| HGG13 | FLGITNML | FAM122A | chr9.68780820.A>T | H13A7 |
| HGG13 | RLQPDRVAM | ACO2 | chr22.41507892.G>A | H13A8 |
| HGG13 | GMVLAEDGV | ALK | chr2.29226954.G>A | H13A9 |
| HGG13 | TVMTITVYA | CEACAM5 | chr19.41715887.C>T | H13A10 |
| HGG13 | IQLAVFFFL | CD34 | NA | H13A11 |
| HGG13 | GLRVVAIQV | FGF13 | chrX.138703039.C>A | H13A12 |
| HGG13 | LLLPAHYETL | CHN2 | chr7.29509358.C>T | H13B1 |
| HGG13 | FCLSPPPTLL | DNAH2 | NA | H13B2 |
| HGG13 | SQIFFSLHL | OR7C1 | NA | H13B3 |
| HGG13 | GTIGLFLPL | CHRNA2 | chr8.27461651.G>A | H13B4 |
| HGG13 | HLEQEEAFL | TRIO | chr5.14369506.A>G | H13B5 |
| HGG13 | SLHLDAWTI | OR7C1 | NA | H13B6 |
| HGG13 | HLDAWTIYS | OR7C1 | NA | H13B7 |
| HGG13 | LLLPAHYET | CHN2 | chr7.29509358.C>T | H13B8 |
| HGG13 | VLHFPLVNC | PLA2G4F | chr15.42142555.G>A | H13B9 |
| HGG13 | FFLGITNML | FAM122A | chr9.68780820.A>T | H13B10 |
| HGG13 | QLAVFFFLL | CD34 | NA | H13B11 |
| HGG13 | LLGFSHLANL | OR10C1 | chr6.29440076.G>A | H13B12 |
| HGG13 | FLTGISQNYA | DNAH3 | chr16.20935410.C>T | H13C1 |
| HGG13 | ALGALLILQL | NOTCH4 | chr6.32199062.C>T | H13C2 |
| HGG13 | IVFPIIVIVV | SLC6A16 | chr19.49293272.C>T | H13C3 |
| HGG13 | YLRLQPDRV | ACO2 | chr22.41507892.G>A | H13C4 |
| HGG13 | YLDATFSPCL | PITRM1 | chr10.3165440.A>G | H13C5 |
| HGG13 | NIYPNIIAM | PTEN | chr10.87864548.T>A | H13C6 |
| HGG13 | AVVGPLPTM | NOTCH2 | chr1.119915724.G>A | H13C7 |
| HGG13 | TLAELVRYYM | PTPN11 | chr12.112450416.A>G | H13C8 |
| HGG13 | VMFIGVNLT | MT-COX1 | chrM.7149.A>G | H13C9 |
| HGG13 | TVMFIGVNL | MT-COX1 | chrM.7149.A>G | H13C10 |
| U87 | SLADLCFSTNV | OR2D2 | Chr11.6913505.T>C | U87 1 |
| U87 | SLADLCFSTNVV | OR2D2 | Chr11.6913505.T>C | U87 2 |
| U87 | TVADFWHMV | PTPN5 | Chr11.18754846.C>T | U87 3 |
| U87 | GILVFFLEV | SLC6A12 | Chr12.313816.G>A | U87 4 |
| U87 | ILVFFLEVAL | SLC6A12 | Chr12.313816.G>A | U87 5 |
| U87 | KELPMFTYRV | MYRF | Chr11.61541446.C>T | U87 6 |
| U87 | STVADFWHMV | PTPN5 | Chr11.18754846.C>T | U87 7 |
| HGG2 | YITAAYVEM | MCM7 | chr7.100094259.T>C | H2C4 WT |
| HGG2 | VLFQCMPVPV | WDR91 | chr7.135208809.C>T | H2D4 WT |
| HGG11 | KLPDLWRK | LAMA3 | chr18.23909188.C>T | H11C4 WT |
| HGG13 | ITEEVWYFL | DNAH3 | chr16.20963718.G>A | H13A2 WT |
| HGG13 | FQGITNML | FAM122A | chr9.68780820.A>T | H13A7 WT |

**Supplementary table 6. Selected HLA-A2 binding neoantigen peptides for investigation**.
